# Dissolved Barium Causes Toxicity to Groundwater Cyclopoida

**DOI:** 10.1002/etc.5956

**Published:** 2024-08-13

**Authors:** Merrin S. Adams, Kitty S. McKnight, David M. Spadaro, Monique T. Binet, Grant C. Hose, Stephen Fenton, Stuart L. Simpson

**Affiliations:** ^1^ CSIRO Environment Lucas Heights New South Wales Australia; ^2^ School of Natural Sciences Macquarie University Macquarie Park New South Wales Australia; ^3^ Chevron Australia Perth Western Australia Australia; ^4^ CSIRO Environment Dutton Park Queensland Australia

**Keywords:** Aquifer, Barite, Copepod, Drilling mud, Stygobiont

## Abstract

Barium (Ba) dissolution and mobilization in groundwater are predominantly controlled by sulfate because of the low solubility of barium sulfate (BaSO_4_) minerals. Naturally present at low concentrations in groundwater, elevated concentrations of Ba can occur as a result of anthropogenic activities, including use of barite in drill operations, and geogenic sources such as leaching from geological formations. No toxicity data exist for Ba with groundwater organisms (stygofauna) to assess the risk of elevated Ba concentrations. The present study measured Ba toxicity to two stygobiont Cyclopoida species: one collected from Wellington and the other from Somersby, New South Wales, Australia. Toxicity was measured as cyclopoid survival over 2, 4, 7, 14, 21, and 28 days in waters of varying sulfate concentration (<1–100 mg SO_4_/L). When sulfate was present, dissolved Ba concentrations decreased rapidly in toxicity test solutions forming a BaSO_4_ precipitate until dissolved sulfate was depleted. Barium in excess of sulfate remained in the dissolved form. The toxicity of Ba to cyclopoids was clearly attributed to dissolved Ba. Precipitated Ba was not toxic to the Wellington cyclopoid species. Toxicity values for dissolved Ba for the Wellington and Somersby cyclopoid species included a (21‐day) no‐effect concentration of 3.3 mg/L and an effective concentration to cause 5% mortality of 4.8 mg/L (at 21 days). Elevated dissolved Ba concentrations due to anthropogenic and/or biogeochemical processes may pose a risk to groundwater organisms. Further toxicity testing with other stygobiont species is recommended to increase the data available to derive a guideline value for Ba that can be used in contaminant risk assessments for groundwaters. *Environ Toxicol Chem* 2024;43:2501–2514. © 2024 The Author(s). *Environmental Toxicology and Chemistry* published by Wiley Periodicals LLC on behalf of SETAC.

## INTRODUCTION

Shallow groundwater comprises approximately 30% of the world's freshwater reserves (Danielopol et al., 2003; Ferguson et al., 2021) and is a resource vital for drinking water and supporting irrigated agriculture and food production in various regions. However, the growing pressures of urban, agricultural, and industrial development pose a risk to groundwater resources and the ecosystems they support (Kretschmer et al., 2023). Anthropogenic activities, including agriculture, groundwater abstraction, oil and/or gas production, and large‐scale mining, have the potential to release anthropogenic and geogenic (resulting from geological processes) contaminants into groundwater (Giri & Singh, 2019; Zhang et al., 2023).

Water quality guidelines are well established for marine and fresh surface waters (Australian and New Zealand Governments [ANZG], 2018; Canadian Council of Ministers of the Environment, 2023; Di Lorenzo et al., 2023; US Environmental Protection Agency, 2023); however, water quality guideline values derived from ecotoxicological studies with surface water species (e.g., *Lemna minor*, *Ceriodaphnia dubia*, and *Danio rerio*) may not be appropriate to protect groundwater organisms (Castaño‐Sánchez et al., 2020; Hose, 2005). Ecotoxicological effects data for contaminant exposures to subterranean species are needed to derive guideline values that can be reliably used in environmental risk assessments to protect the unique groundwater species and their ecosystem functions (Di Lorenzo et al., 2019; Hose, 2005, 2007).

Groundwater ecosystems host a diversity of biota, including microbes, fungi, protozoa, and higher orders such as nematodes, worms, arthropods, predominantly crustaceans, and occasionally vertebrates (e.g., eels and fish; Marmonier et al., 2023). The higher‐order organisms, collectively referred to as *stygofauna*, are increasingly being recognized as a diverse and ecologically significant component of aquifers that provides ecosystem services such as nutrient cycling and maintaining water quality and flow (Griebler et al., 2019; Mermillod‐Blondin et al., 2023). Obligate groundwater fauna (stygobionts) are often highly adapted to the groundwater environment and have morphological and physiological characteristics that differ from surface water species due to their unique evolution and living environment (Canivet & Gibert, 2002; Danielopol et al., 1994; Humphreys, 2008). Morphological adaptations arise from restricted space, constant darkness, stable temperatures, low oxygen, food scarcity, slow water recharge, and limited connection to neighboring groundwater communities (Marmonier et al., 2023). Physiological responses include low metabolic rates, low reproductive rates, and longer life cycles compared to similar surface‐water species, as well as other traits such as their small size, worm‐like body shapes, and lack of eyes and pigmentation (Hose et al., 2023).

Knowledge of the toxicity of contaminants to stygobionts is limited. Groote Woortmann et al. (2024) summarize 46 studies of the response of stygofauna to stressors that include metals, pesticides, organic compounds, and temperature change. Toxicity testing has been undertaken on stygobionts including Oligochaeta, Amphipoda, Isopoda, Syncarida, Harpacticoida, and Cyclopoida, although not all taxa were tested with each contaminant. Survival has been the toxicity endpoint measured most frequently with typically short exposure durations (48 and 96 h; Groote Woortmann et al., 2024), although longer exposure durations (14–28 days) are recommended (Di Lorenzo et al., 2019). Too few data exist to determine if stygobiont species have overall a higher, lower, or similar sensitivity to contaminants compared to their surface‐water counterparts (Castaño‐Sánchez et al., 2020; Hose et al., 2019); however, stygobionts are at least as sensitive as surface‐water species to some contaminants. For example, the concentrations of arsenic (III), chromium (VI), and zinc that cause 10% mortality (14−28‐day LC10s) to stygobiont crustaceans were close to or below the guideline concentration designated to protect 95% of surface‐water species (Supporting Information, Table S1; ANZG, 2018). Guideline values based on the sensitivity of stygofauna to contaminants will provide greater certainty when establishing the risk of contaminants to groundwater ecosystems.

Barium is naturally found in groundwater at low concentrations (Shand & Edmunds, 2008) due to the low solubility of minerals such as barite (BaSO_4_) and witherite (BaCO_3_). For example, the median concentration of dissolved Ba in European groundwaters was 47 µg/L, and concentrations above several hundred micrograms of Ba per liter in natural groundwater are rare (Shand & Edmunds, 2008). Barite is predominantly extracted for use in the oil and gas industry because it is a major component in drilling fluids (i.e., acts as a weighting agent to prevent blowouts when drilling wells; US Geological Survey, 2018); however, it can enter the environment via infiltration into groundwater. Barite dams, created to contain used drilling muds, are also sources of Ba in groundwater via seepage. For example, dissolved Ba concentrations in groundwater within the vicinity of oil and gas extraction operations in the Yan'an area of northwest China ranged from 0.01 to 45 mg/L (with an average of 3 mg/L, *n* = 36; Zhang et al., 2023). Geogenic sources of Ba can arise as a result of changes in geochemistry, especially those that influence Ba solubility (Agency for Toxic Substances and Disease Registry, 2007).

The speciation of Ba is determined by its solubility in the presence of major ions, predominantly sulfate followed by carbonate (Golding et al., 2018). In the presence of sulfate, dissolved Ba forms a stable barium sulfate (BaSO_4_) precipitate (solubility limit of ~2.5 mg/L, equivalent to 1.5 mg Ba/L; Aylward & Findlay, 2008). When all of the sulfate has been consumed, a barium carbonate (BaCO_3_) precipitate forms (solubility limit of ~20 mg/L, equivalent to 14 mg Ba/L; Aylward & Findlay, 2008), with excess Ba remaining in the dissolved phase (Golding et al., 2018). Sulfate plays a role in Ba dissolution in groundwater, and it is the dominant factor controlling Ba solubility and transport in groundwater (Vilcáez, 2020).

Ecotoxicity data have not been derived for Ba using stygobionts, although some studies exist for surface‐water crustaceans (Verbruggen et al., 2020). The quality of those studies varies considerably and is related to the extent (or lack) of characterization of Ba speciation and the composition of the toxicity test solutions, specifically sulfate (Verbruggen et al., 2020). Some of the studies have highlighted that different forms of Ba differ in their toxicity to aquatic organisms (Golding et al., 2018). For example, the mortality of Cladocera was attributed to dissolved Ba and not precipitated BaSO_4_; however, precipitated BaSO_4_ was more toxic than dissolved Ba to a microalga (Golding et al., 2018).

The aims of the present study were to (i) describe detailed ecotoxicity methods for testing Ba with stygofauna, (ii) derive toxicity values for Ba using stygobiont Cyclopoida, and (iii) identify if the observed toxicity could be attributed to dissolved or precipitated Ba or a combination of the two. Cyclopoids were selected for the present study because they have been successfully used in ecotoxicity tests and are sensitive to metal contaminants (Hose et al., 2016). They are also readily available in sufficient numbers from local reference (unimpacted) sites. The results of the present study also inform future ecotoxicity studies of Ba toxicity to other stygobiont taxa, which is necessary to derive groundwater quality guideline values for use in environmental risk assessments.

## METHODS

### Stygofauna and groundwater collections

Stygofauna and associated groundwaters were collected from bore WRS05 at Wellington, New South Wales (NSW; 32°34′S, 148°59′E, bore depth = 23 m) on three occasions: April 11, 2021, April 20, 2022, and August 10, 2022. Stygofauna were also collected from bore GW075039 at Somersby, NSW (33°22′15.4″S, 151°18′09″E, bore depth = 29 m) on October 22, 2020. The Wellington site is an alluvial aquifer associated with the Macquarie River, and the Somersby site is a fractured sandstone aquifer.

The copepods used in the present study are undescribed species of the family Cyclopidae. Taxonomy of the copepods from Somersby was determined by sequencing the mitochondrial cytochrome oxidase 1 gene (see Hose et al., 2016). These copepods were identified as a single clade (accession number KX244839) that match most closely to *Mesocyclops* spp. Copepods from Wellington were identified morphologically as *Diacyclops* sp.

Stygofauna were collected using a motorized inertia pump (Waterra) (Hose & Lategan, 2012). Approximately 200 L of groundwater was pumped and passed through a 63‐µm mesh sieve (stainless steel) to collect the invertebrates. The sieve contents were placed in a sealable, 1‐L plastic container that was filled to the brim with groundwater (filtered through a 63‐µm mesh sieve) and placed in a portable cooler for transportation to the laboratory. A further 20 L of groundwater was pumped, filtered (63‐µm mesh sieve), and collected into a polypropylene carboy. The temperature of the water at the time of collection was 18 °C, except for the Wellington collection on April 20, 2022, when it was 21 °C. Stygofauna and water were transported by car to the testing laboratory on the same day of collection. Stygofauna were placed in a temperature‐controlled cabinet at 18 °C in the dark with the container lid loosened to allow for air exchange. The filtered (<63‐µm sieve) water was stored at 4 °C in the dark. Groundwater with low sulfate concentration was also collected from bores in the Pilbara region, Western Australia, for use in toxicity tests (referred to as *low‐SO*_*4*_).

### Preparation of waters for toxicity testing

Subsamples of toxicity test waters (groundwater) were decanted into an acid‐washed 2‐L glass bottle and placed in the incubator (18 °C) to equilibrate for 1 to 3 days prior to the start of a toxicity test. Precipitates were noticeable in some of the groundwaters upon saturation with oxygen. Equilibrated groundwaters used in toxicity tests were decanted immediately prior to use, which was done carefully to avoid entraining precipitated particulates.

Stock solutions of Ba were prepared volumetrically by dissolving BaCl_2_ · 2H_2_O (analytical reagent grade; Sigma‐Aldrich; solubility 370 g/L; Aylward & Findlay, 2008) in high‐purity water (18 MΩ · cm Milli‐Q water; Millipore). A range of stock solutions were prepared (10–10,000 mg Ba/L). Dissolved Ba concentrations were within 8% of the nominal concentration.

Groundwaters used in toxicity tests are described in Table 1. Three additional water preparations were used to create different fractions of dissolved and precipitated Ba (Table 1): standard preparation plus filtration to <0.45 µm (W2); standard preparation with equilibration for 88 h, followed by filtration to <0.45 µm (W3); and a sulfate‐free synthetic hardwater (W4). The sulfate‐free water replicated the water hardness and pH of groundwater from Wellington (~430 mg CaCO_3_/L and pH 7.4 − 7.7). The sulfate‐free synthetic water was prepared by dissolving 0.258 g CaCl_2_ · 2H_2_O, 0.541 g MgCl_2_ · 6H_2_O, and 0.020 g KCl in high‐purity water overnight, along with 0.486 g NaHCO_3_ dissolved in a separate beaker (Golding et al., 2018). The dissolved salts were then made up to 1 L using high‐purity water, filtered to 0.45 µm, with pH adjusted to 7.4 to 7.7 by the dropwise addition of 6 M HCl.

**Table 1:** Preparation of groundwaters and sulfate‐free synthetic water used in toxicity tests

Label	Site/collection	Preparation of dilution water	Expected characteristics
S	Somersby, 2020	Standard preparation: Groundwater (decanted to remove settled precipitates) with BaCl_2_ stock added	Dissolved and potentially small quantity of precipitated Ba present (due to expected low sulfate concentration)
W	Wellington, 2021	Standard preparation: Groundwater (decanted to remove settled precipitates) with BaCl_2_ stock added	Dissolved and precipitated Ba present
			Decreasing dissolved and increasing precipitated Ba with increasing toxicity test duration (generally rapid up to 96 h before slowing/stopping)
W‐C1	Wellington, 2022, Collection 1	Standard preparation: Groundwater (decanted to remove settled precipitates) with BaCl_2_ stock added	Dissolved and precipitated Ba present
			Decreasing dissolved and increasing precipitated Ba with increasing toxicity test duration (generally rapid up to 96 h before slowing/stopping)
W1	Wellington, 2022, Collection 2	Standard preparation: Groundwater (decanted to remove settled precipitates) with BaCl_2_ stock added	Dissolved and precipitated Ba present
			Decreasing dissolved and increasing precipitated Ba with increasing toxicity test duration (generally rapid up to 96 h before slowing/stopping)
W2	Wellington, 2022, Collection 2	Groundwater (decanted to remove settled precipitates) filtered to 0.45 µm,^a^ followed by addition of BaCl_2_ stock	Dissolved and precipitated Ba present
			Decreasing dissolved and increasing precipitated Ba with increasing toxicity test duration (generally rapid up to 96 h before slowing/leveling)
			No natural groundwater particulates (>0.45 µm)
			Possibly lack of food sources (>0.45 µm)
W3	Wellington, 2022, Collection 2	Groundwater (decanted to remove settled precipitates) with BaCl_2_ stock added, incubated for 88 h at 18 °C in the dark, then filtered to 0.45 µm	Dissolved Ba only, stable concentrations over time
			No Ba precipitates
			No natural groundwater particulates (>0.45 µm)
			Possible lack of food sources (>0.45 µm)
W4	Not applicable	Sulfate‐free synthetic hard water (~430 mg CaCO_3_/L, pH 7.4−7.7) filtered to 0.45 µm, then BaCl_2_ stock added	Dissolved Ba only, stable concentrations over time
			No Ba precipitates
			No particulates (>0.45 µm)
			No food sources
Low‐SO_4_	Pilbara region, 2022	Low‐sulfate groundwater ‐ Standard preparation: Groundwater (decanted to remove settled precipitates) with BaCl_2_ stock added	Dissolved and small quantity of precipitated Ba present (due to expected low sulfate concentration)

^a^ Filtered to 0.45 µm using an acid‐washed nitrocellulose membrane and acid‐washed polycarbonate filtration unit.

### Chemical analysis

Groundwaters and the synthetic water were analyzed for a range of chemical and physicochemical parameters by CSIRO Environment in 2020 and 2021, and the National Measurement Institute 2022. Analytes and methods are detailed in Supporting Information, Table S2. Analysis of each analyte included quality controls consisting of blanks, duplicate analyses, and spiked recoveries for each batch of samples. Selected groundwaters were reanalyzed to identify if the groundwater composition remained stable during storage (4 °C, dark) over the 3‐year study. Temperature, electrical conductivity (Mettler Toledo Seven Go Duo Pro with InLab probe), dissolved oxygen (as milligrams per liter and percentage saturation; WTW Multi 3410 with WTW FDO®925 probe), and pH (Model 420 with Hanna HI2031 probe; Thermo Orion) were also measured in toxicity tests. The probes were calibrated following the manufacturers' protocols.

Concentrations of dissolved Ba in toxicity tests were measured by CSIRO Environment. For each toxicity test, additional well plates were prepared (without animals) and incubated at 18 °C, and subsamples were taken for chemical analysis on days 0, 2, 4, 7, 14, 21, and 28. At each time point, test solutions were diluted with high‐purity water, then filtered (within 2–10 min) through a 0.45‐µm filter membrane (polyethersulfone, Minisart®; Sartorius) using a plastic syringe into a 5‐mL polypropylene vial containing 100 µL nitric acid (Suprapur; Merck; final HNO_3_ concentration of 2% v/v). Ideally, filtration before dilution would ensure any precipitated Ba in the subsample did not redissolve before preservation with acid. In the present study, additional experiments confirmed that filtration within 15 min of dilution did not result in the dissolution of any BaSO_4_ precipitate before filtration (data not shown), indicating that the dissolved Ba concentrations measured in the present study were accurate representations of the dissolved concentrations in toxicity test solutions. Control treatments were prepared to achieve a final dilution of 1 in 5. Barium treatments were prepared to achieve a final dilution of 1 in 50 or 1 in 500, depending on the total added Ba and the expected dissolved concentration.

Samples were analyzed by inductively coupled plasma‐atomic emission spectroscopy (ICP‐AES) within 1 to 3 weeks using calibration standards of 0, 0.5, 1, 2, 4, and 10 mg Ba/L at a wavelength of 455 nm, where there are no known interferences in the optical signal from other elements. A drift standard of 1 mg Ba/L was analyzed every 10 samples along with a blank (Milli‐Q water). Measured concentrations of the drift standard throughout the analysis remained within 2% of the nominal concentration. Measured Ba concentrations in the blanks fluctuated within 5% of the limit of detection. Test solutions were diluted to within the ICP‐AES instrument calibration range (<10 mg Ba/L). The limit of detection was approximately 1 µg Ba/L, with the exact detection limit varying slightly with each batch of samples analyzed.

Total Ba concentrations could not be measured because BaSO_4_ (or BaCO_3_) precipitates that formed either adhered to the walls of the test container (i.e., unable to take a representative homogenous subsample for analysis) or were insoluble in standard acid digest solutions (HCl, HNO_3_). Therefore, total Ba concentrations were reported as the nominal Ba concentration added.

### Toxicity tests

Eight toxicity tests were carried out using adult cyclopoids and waters from different locations as outlined in Supporting Information, Table S3. Survival of cyclopoids was recorded over 28 days after exposure to Ba following the methods described in Hose et al. (2016). Cyclopoids were tested in their native groundwater collected at the same time as the animals. The exceptions were for tests with low‐SO_4_ groundwater and sulfate‐free synthetic water, which both used cyclopoids from the second collection at Wellington in 2022.

Individual cyclopoids were transferred from a mixed stygofauna population into a small Petri dish containing a few milliliters of groundwater as a preisolation step. Untreated 24‐well plates (Greiner bio‐one; CELSTAR®) were labeled with control and Ba treatments randomized within and across the required number of well plates. Two milliliters of test water (e.g., groundwater, synthetic water) was added to each test well. An individual cyclopoid was then transferred into each well using a 200‐µL pipette (with the end of the pipette tip sawn off and replaced between treatments to avoid cross‐contamination). Approximately 20 µL of associated groundwater was transferred with each animal. Barium was added to each treatment well using the barium chloride (BaCl_2_) stock solutions and ensuring that the volume added did not exceed 200 µL (10% of total volume). Well plates were placed in a plastic tub (with lid) containing beakers holding high‐purity water. This maintained humidity and minimized evaporation from the well plates. The tubs were then placed in an environmental cabinet, in the dark at 18 ± 1 °C. Replicates of control and each Ba treatment varied between tests depending on the number of cyclopoids available for testing on the day. Generally, 8 to 14 replicates were tested for each Ba treatment and control. Total (added) Ba concentrations ranged from 0.5 to 2560 mg Ba/L.

Additional well plates were prepared as described above, without the addition of animals, and used for measuring dissolved Ba concentrations and water quality parameters. Subsamples for dissolved Ba analysis were taken within 15 min of adding the Ba stock solution, representing the dissolved Ba concentration at the beginning of the test (0 h, or day 0). Subsamples were also taken on days 2, 4, 7, 14, 21, and 28. Measurements of pH, conductivity, and dissolved oxygen were taken at the beginning of the test and after 28 days.

Cyclopoids were observed under a light microscope (Olympus SZX10) and classified as alive or dead after 2, 4, 7, 14, 21, and 28 days. Animals were shielded from light by covering the well plates when not being observed. Survival was defined as any movement of body or sensory appendages within 15 s. If necessary, gentle prodding encouraged movement when viewed under a microscope. Assessments of cyclopoid survival occurred in every well at all time points, even those previously classified as dead because in some instances organisms were initially misclassified as dead because of their inherent lack of movement. This provided certainty that mortality rather than dormancy or immobility was measured (Hose et al., 2016). Mortality in the control should be <20% for test acceptability (Di Lorenzo et al., 2019). The present study also reports results where this criterion was not met to enhance the data available to assess the effects of Ba on longer‐term exposures to Ba.

Deviations from the above methods were carried out in 2021 and 2020 as test methods and techniques were streamlined over time. In 2020, the cyclopoids were individually transferred from the collection container directly into individual wells without a preisolation step. Earlier toxicity tests also adjusted the volume of test water added (e.g., 1.8 mL) to account for the volume of groundwater associated with the animal and the volume of Ba stock solution added to each well to achieve a total final volume of 2 mL. Adopting a standard test water volume of 2 mL streamlined the test setup to minimize stress on the field‐collected cyclopoids.

### Statistical analysis

#### Preparation of data for statistical analysis

Decreasing concentrations of dissolved Ba in test solutions over time were integrated to calculate a time‐weighted‐average (TWA) exposure concentration for the period prior to each observation time point (2, 4, 7, 14, 21, and 28 days) using Equation (1):

(1) $$\text{TWA}=\frac{\sum_{i=1}^{1-6}C_{i}T_{i}}{\sum_{i=1}^{1-6}T_{i}}$$

In Equation (1), TWA is time‐weighted average concentration of dissolved Ba at time period *T_i_*, *i* is time period (1 = 0–2, 2 = 2–4, 3 = 4–7, 4 = 7–14, 5 = 14–21, and 6 = 21–28 days), *C*_*i*_ is concentration of dissolved Ba (milligrams per liter) at the start of the time period (*i*) minus concentration of dissolved Ba concentration at the end of the time period (*i*), and *T*_*i*_ is time in days at the end of the time period (*i*) minus time in days at the start of the time period (*i*); where concentrations of dissolved Ba remained stable over the 28‐day test duration, the 28‐day average (*n* = 6 or 7) was used as the exposure concentration for all time points. This occurred in waters with ≤1 mg/L sulfate.

The proportion of surviving cyclopoids was reported for each control and Ba treatment within each toxicity test. The proportion of surviving cyclopoids as a percentage of the control proportion was also calculated. This allowed for normalization of cyclopoid survival between toxicity tests when cyclopoid survival was <100%.

#### Derivation of toxicity values (effect and no‐effect concentrations)

The concentration of Ba to cause an *x*% effect on cyclopoid survival (EC*x*, where *x* = 5%, 10%, 20%, and 50%, along with their 95% confidence limits) and the no‐effect concentration (NEC) on cyclopoid survival were derived using the drc package (Ritz et al., 2015) in RStudio (Version 2024.4.2.764; Posit Team, 2024).

Curve‐fitting of individual and/or combined data sets was carried out using survival data with and without normalization to the proportion of surviving cyclopoids in the control treatment. Control treatments were represented as the measured dissolved concentration and zero for modeling dissolved and total (added) concentration–response curves, respectively. Five curve‐fitting models were assessed for goodness of fit to derive EC*x* values: Weibull Type 2, Weibull Type 1, log‐logistic, log‐normal, and Gompertz. Three‐parameter models were used with the upper limit fixed at the control response (proportion of stygofauna alive), the lower limit fixed at zero (default), and the type of data classified as binomial. This is effectively a two‐parameter model, the preferred type for binomial data. The best‐modeled curve fit was then selected for each data set based on Akaike's information criterion (AIC) and visual observation of individual curves to the data points. Where AIC values and curve fits were similar or there was a lack of data points, professional best judgment was used to select the most appropriate curve‐fit model to derive EC*x* values. The NEC was also derived when concentration–response curves showed a threshold or tipping point using the NEC.2 function. This mainly occurred when multiple data sets were combined.

Toxicity values (EC*x*, NEC) were derived for 2‐, 4‐, 7‐, 14‐, 21‐, and 28‐day exposures. Comparisons of curve‐fit models were made using a one‐way analysis of variance. Residuals were also plotted to observe if the curve fits were biased or evenly distributed using RStudio (Posit Team, 2024).

## RESULTS

### Chemical characterization of waters for toxicity testing

Selected water quality parameters are shown in Table 2, and detailed analysis of parameters (major ion, metals, C, N, and P compounds) is provided in Supporting Information, Table S4. Groundwater from Somersby was lower in pH, conductivity, alkalinity, hardness, and dissolved organic carbon (DOC) compared to groundwater collected from Wellington in 2022. Groundwater from Wellington in 2021 had lower pH, conductivity, and sulfate concentration compared to the two groundwaters collected in 2022, while alkalinity and hardness were higher. Conductivity and DOC in the low‐SO_4_ and sulfate‐free waters also differed from Wellington groundwaters. The groundwaters collected provided sulfate concentrations of 1, 7.4, 22, and 100 mg SO_4_/L for Somersby, low‐SO_4_, Wellington‐2021, and Wellington‐2022, respectively (Table 2). The synthetic water did not contain sulfate (<0.1 mg SO_4_/L). Characteristics of the low‐SO_4_ groundwater aligned with Wellington‐2022 groundwater, except for the sulfate concentration. The sulfate‐free synthetic water had a similar hardness and pH to Wellington‐2022 groundwaters.

**Table 2:** Summary of the characterization of groundwaters and sulfate‐free synthetic water

			Wellington‐2022			Sulfate‐free
Parameter	Somersby‐2020	Wellington‐2021	Collection 1	Collection 2 (W1)	Low‐SO_4_	hard water (W4)
Sulfate (mg/L)	1	22^a^	89^b^	110^b^	7.4	<0.1
Alkalinity (mg/L)	<10	370	300	310	320	240
Hardness (mg/L)	22	445	428	424	308	437
pH	4.6	4.7	7.7	7.4	7.6	7.9
Conductivity (µS/cm)	150	990	1090	1100	783	1500
DOC	<1	2.0	2.4	1.9	0.8	<0.5
Ba, dissolved (µg/L)	7.5	16	85	80	41	<1
Sr, dissolved (µg/L)	11	726	710	760	200	<1
B, dissolved (µg/L)	15	37	37	24	88	<5
Cu, dissolved (µg/L)	2	2	<1	<1	<1	<1
Ni, dissolved (µg/L)	1	4	<1	<1	<1	1.7
Zn, dissolved (µg/L)	13	7	<1	<1	1.3	<1

^a^ Reanalysis after 17‐month storage (4 °C in the dark) was 65 mg/L sulfate.

^b^ An average of 100 mg/L sulfate was used for Wellington‐2022 when results from Collection 1 and Collection 2 were combined.

DOC = dissolved organic carbon.

Dissolved Ba concentrations in the field‐collected groundwater ranged from 7.5 to 85 µg/L, while concentrations of many metals were <1 µg/L (e.g., Cd, Co, Cr, Mo; Supporting Information, Table S4). Dissolved concentrations of the alkaline metal strontium ranged from 11 to 760 µg/L. The influence of strontium concentrations on aquatic biota is difficult to assess because of a lack of local freshwater guideline values. Based on a comparison to the freshwater guideline value of 2.5 mg/L published in Canada (Environment and Climate Change Canada, 2020), the concentrations of strontium in the groundwaters are not expected to confound the toxicity results for Ba obtained in the present study.

### Survival of cyclopoids in control treatments

**Figure 1:**
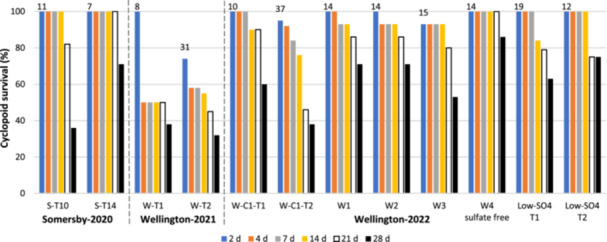
Survival of stygobiont cyclopoids after 2–28 days in groundwater and a sulfate‐free synthetic water (control water with no added Ba). Cyclopoids were collected from Somersby in 2020 and Wellington in 2021 and twice in 2022. Cyclopoids were tested in their native groundwater. Wellington‐2022 cyclopoids were also tested in a low‐SO_4_ groundwater on two occasions. The value above the blue column is the total number of cyclopoids in each test. C = collection number; T = test number; W = Wellington‐2022 cyclopoids (Collection 2) tested in unfiltered native groundwater (W1), filtered (W2), preequilibrated (88 h) and filtered (W3), and a filtered synthetic sulfate‐free hardwater (W4).

Survival of cyclopoids in the control waters (no added Ba) decreased over 28 days, with the rates varying between toxicity tests (Figure 1; Supporting Information, Table S5). Cyclopoids from Somersby had the highest survival rates in their native groundwater, with >80% survival up to 21 days (*n* = 2 toxicity tests). Cyclopoids from Wellington collected in 2021, also in their native groundwater, had the lowest survival rates (*n* = 2), with only the 2‐day exposure for one of two toxicity tests exceeding the recommended acceptable criterion of 80%. Survival was very poor for all other exposure durations (≤58% survival for ≥4 days). Survival of cyclopoids collected from Wellington in 2022 was higher than for cyclopoids collected in 2021, with ≥80% survival for test durations up to 14 days for six of seven tests. Low‐SO_4_ groundwater resulted in similar cyclopoid survival (>80% survival for ≤14 days, *n* = 2). Overall, cyclopoid survival was poor in all groundwaters after 28 days (32%–75%). The exception was for tests in synthetic water, which provided the highest cyclopoid survival rates and met test acceptability criteria for all time points with 100% survival up to 21 days and 86% survival after 28 days. With very poor survival observed for cyclopoids collected from Wellington in 2021, Ba toxicity data from 2021 were not used in the present study to define recommended Ba toxicity values.

### Dissolved Ba concentrations in toxicity tests

**Figure 2:**
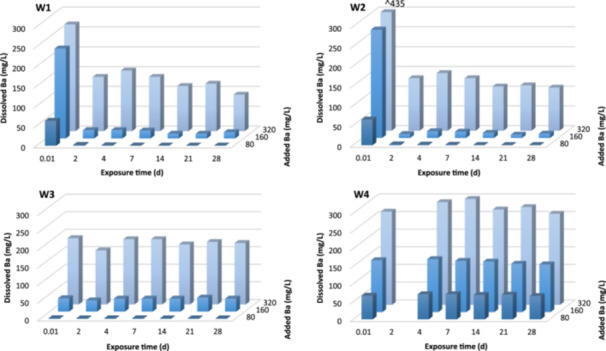
Concentration of dissolved (0.45 µm filtered) Ba over 28 days in different waters. The *z*‐axis describes the Ba treatment concentrations as the nominal added Ba (i.e., total). W1 = Wellington‐2022 (Collection 2) groundwater following decanting to remove particulates; W2 = Wellington‐2022 (Collection 2) groundwater after filtration to 0.45 µm; W3 = Wellington‐2022 (Collection 2) groundwater after Ba equilibration at 18 °C for 88 h followed by filtration to 0.45 µm; W4 = sulfate‐free synthetic water.

Concentrations of dissolved Ba in all of the toxicity test solutions are presented in Supporting Information, Figures S1–S4 and Tables S6–S9, with a summary described here and in Figure 2. Dissolved Ba concentrations remained stable over the 28‐day toxicity tests in sulfate‐free synthetic water and Somersby groundwater (<0.1 and 1 mg/L sulfate, respectively; Figure 2, W4; Supporting Information, Figure S1, respectively), indicating that these two water types provided an exposure to dissolved Ba only (no Ba precipitate). In Wellington and low‐SO_4_ groundwater (Supporting Information, Figures S2–S4), dissolved Ba concentrations rapidly decreased and remained low (<1–2 mg/L) until sulfate was depleted from the water. Excess Ba remained soluble, with dissolved Ba concentrations observed to increase proportionally with increasing total Ba concentrations. Over the 28‐day toxicity tests, dissolved Ba concentrations decreased slightly and slowly (Supporting Information, Figures S2–S4 and Tables S7–S9). The maximum quantity of Ba precipitate formed in the test treatments increased with increasing sulfate concentration in groundwaters (7.4, 22, 100 mg SO_4_/L). Barium precipitate was clearly visible in the test solutions (Supporting Information, Figure S5) as a fine white solid that was easily resuspended.

Filtration of Wellington‐2022 groundwater (W2) did not alter the dissolved Ba concentrations (or extent of precipitated BaSO_4_) over 28 days compared to unfiltered groundwater (Figure 2, W1 and W2). Preequilibration of added BaCl_2_ in Wellington groundwater for 88 h followed by filtration to remove the BaSO_4_ precipitate (W3) resulted in test waters with a stable dissolved Ba concentration over 28‐day toxicity tests (i.e., devoid of detectable precipitated Ba; Figure 2, W3). This was also the case when BaCl_2_ was added to sulfate‐free synthetic water (Figure 2, W4).

Concentrations of dissolved Ba in control treatments over 28 days averaged 0.01 mg/L for Somersby groundwater and 0.04 mg/L for the sulfate‐free synthetic water. Wellington and low‐SO_4_ groundwaters did not exceed TWA dissolved Ba concentrations of 0.09 and 0.05 mg/L, respectively.

### Toxicity of dissolved versus precipitated Ba to Wellington cyclopoids

The toxicity of dissolved‐only and dissolved and precipitated Ba was compared using the same collection of cyclopoids (Wellington‐2022, Collection 2) within the same toxicity test. This eliminated any potential variability due to different cyclopoid isolates and survival rates between collections and toxicity tests. Toxicity data for W3 (Ba precipitate removed) and W4 (sulfate‐free water) were combined because both waters represented exposures to stable dissolved Ba concentrations throughout the 28‐day toxicity test (Figure 2, W3 and W4). In contrast, W1 and W2 with unfiltered and filtered groundwater (110 mg SO_4_/L), respectively, contained observable Ba precipitates (Supporting Information, Figure S5). A low‐SO_4_ groundwater with fewer Ba precipitates was also included in the comparison because it was tested at the same time and with the same collection of cyclopoids.

**Figure 3:**
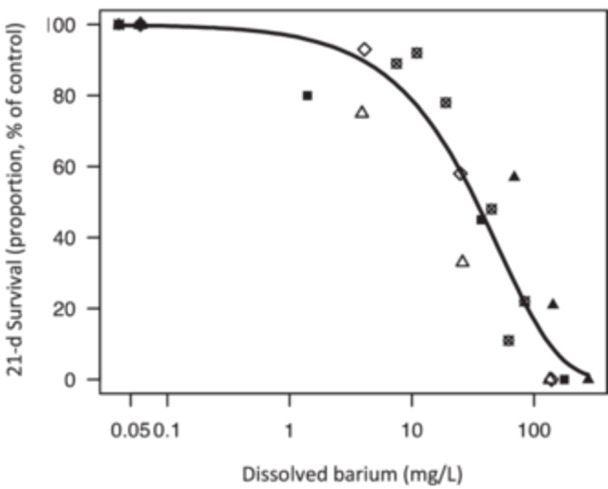
Survival of cyclopoids collected from Wellington‐2022 (Collection 2) in different waters as a percentage of the control. Exposure concentrations are expressed as time‐weighted average for dissolved plus precipitated Ba (open and partially open data points) or 28‐day average dissolved (0.45 μm) Ba concentrations for dissolved‐only Ba (solid data points). The solid line shows the Weibull Type 1 modeled curve fit to all data points. The test exposures were prepared by adding dissolved BaCl_2_ to groundwater with 110 mg SO_4_/L following decanting to remove particulates (W1 [Δ]: dissolved and precipitated Ba) and after filtration to 0.45 µm (W2 [◇]: dissolved and precipitated Ba). Groundwater with a dissolved‐only Ba exposure was prepared by adding dissolved BaCl_2_ to Wellington groundwater, equilibrated at 18 °C for 88 h then filtered to 0.45 µm (W3 [■]: dissolved‐only Ba). Dissolved BaCl_2_ was also added to sulfate‐free synthetic hardwater (W4 [▲]: dissolved‐only Ba). Groundwater with a low sulfate concentration (7 mg SO_4_/L) was used to prepare a dissolved and precipitated Ba exposure with a lower proportion of insoluble precipitated Ba particles (low‐SO_4_ [⊠]).

When expressed as dissolved Ba (average or TWA), survival of cyclopoids exposed to dissolved and precipitated Ba in W1 (unfiltered), W2 (filtered), and low‐SO_4_ groundwater followed a similar concentration–response relationship to that observed for dissolved‐only exposures of Ba in W3 and W4 (Figure 3). Removal of W3 and W4 (dissolved‐only Ba exposures) did not significantly alter the modeled curve fit (*p* > 0.05). This was also observed for all other test durations of 2 to 28 days (Supporting Information, Figure S6 and Table S10). These results indicate that the toxicity of Ba to cyclopoids was due to dissolved Ba exposures and not insoluble precipitated BaSO_4_.

The thresholds for different levels of toxicity (e.g., EC10, EC5) derived using the combined dissolved Ba data set (W1, W2, W3, W4, and low‐SO_4_) decreased with increasing exposure duration (Supporting Information, Table S11) with a 4‐day EC10 of 28 (14–43) mg/L and a 21‐day EC10 of 3.9 (0.39–7.4) mg/L (Supporting Information, Table S11).

### Ba toxicity to Wellington and Somersby cyclopoids as total and dissolved concentrations

**Figure 4:**
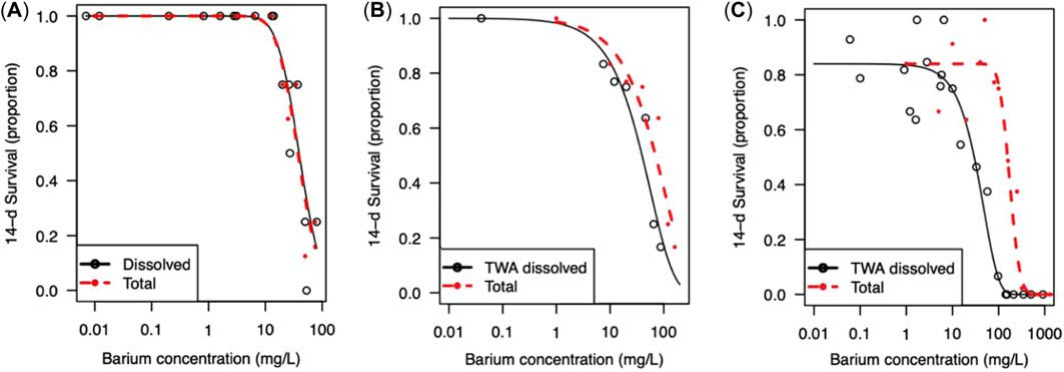
Toxicity of Ba to cyclopoids after a 14‐day exposure. (**A**) Somersby cyclopoids in Somersby groundwater containing 1 mg SO_4_/L; (**B**) Wellington‐2022 cyclopoids in low‐SO_4_ groundwater with 7 mg SO_4_/L; (**C**) Wellington‐2022 cyclopoids in Wellington‐2022 groundwater with an average of 100 mg SO_4_/L (data from two collections of cyclopoids and groundwater with 89 and 110 mg SO_4_/L, respectively). Toxicity is represented as total (red) and dissolved (black) average or 14‐day time‐weighted average concentrations of Ba. TWA = time‐weighted average.

Toxicity data from all of the individual toxicity tests in the present study are summarized in Supporting Information, Tables S6, S8, and S9. Concentration–response relationships for 14‐day survival of cyclopoids expressed as total and dissolved Ba are shown for waters with different sulfate concentrations of 1, 7, and 100 mg/L (Figure 4). The 14‐day data were used in the present study because survival in all of the control treatments met test acceptability criteria. The concentration–response relationships for Somersby cyclopoids in groundwater containing 1 mg SO_4_/L were the same when expressed as total and dissolved Ba concentrations (Figure 4A). This was expected because chemical analysis confirmed that all of the Ba was in the dissolved form. As sulfate concentrations in groundwater increased, toxicity expressed as total Ba shifted to the right (less toxic), as shown for groundwaters with 7 and 100 mg SO_4_/L (Figure 4B,C, respectively). The same trend between dissolved and total Ba concentration–response curves was observed for all other test durations (2–28 days), even when control data did not reach 80% survival (Supporting Information, Figures S7–S9).

**Figure 5:**
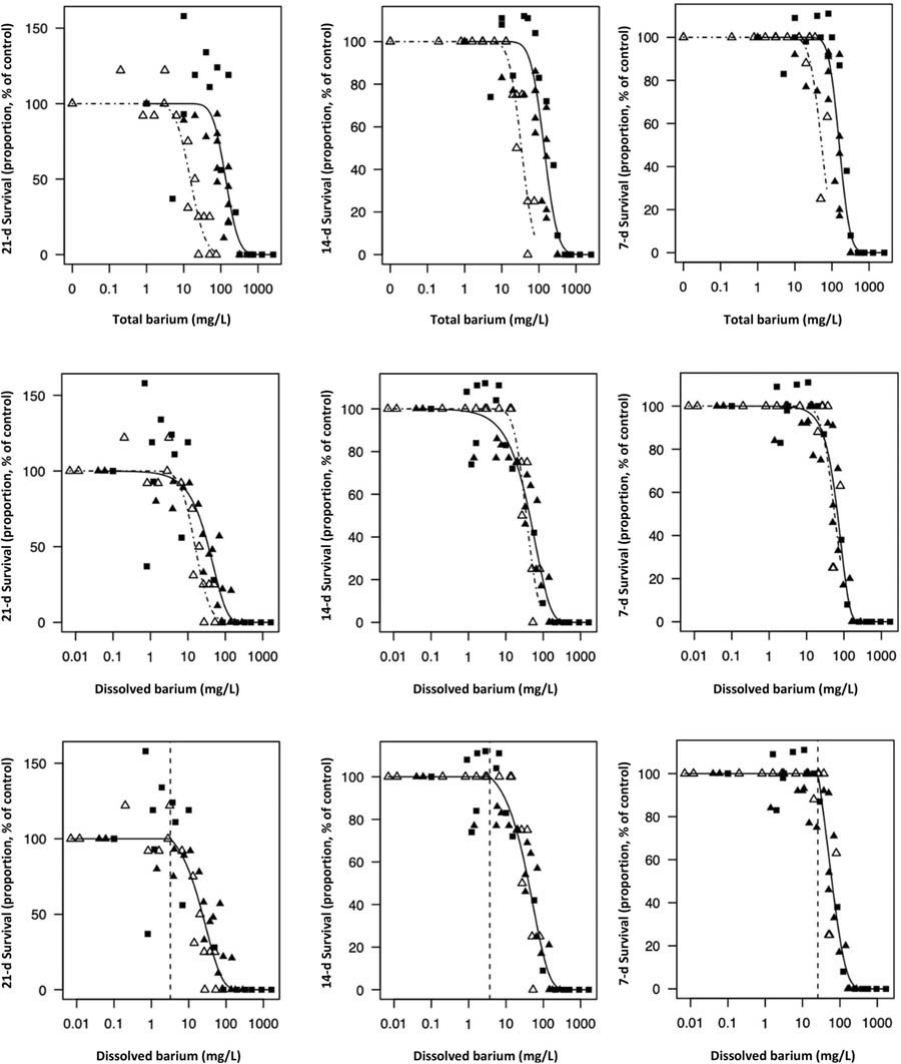
Concentration–response curves for cyclopoid survival after 21‐, 14‐, and 7‐day exposures to Ba, as total (nominal added Ba) or dissolved Ba (average or time‐weighted average). Data points represent the proportion of surviving cyclopoids from Wellington (Collection 1 = solid square; Collection 2 = solid triangle) and Somersby (open triangle) for all water types tested. Top row shows total Ba with log‐normal curve fits for Wellington (solid) and Somersby (dashed) cyclopoids. Middle row shows dissolved Ba with Weibull Type 1 and log‐normal curve fits for Wellington (solid) and Somersby (dashed) cyclopoids, respectively. Bottom row shows dissolved Ba with no‐effect concentration (NEC) curve fits (solid) for the combined Wellington and Somersby cyclopoid data set and the NEC value (dashed line).

The toxicity of dissolved Ba appears to be similar for the two cyclopoid species (Somersby and Wellington‐2022 species), with observable mortality occurring at approximately 10 to 100 mg/L (Figure 4). This was further explored by comparing the concentration–response relationships between total and dissolved Ba for the two species for all water types tested (Figure 5). The concentration–response curves based on total Ba concentration for Wellington‐2022 cyclopoids sits to the left of the curve for Somersby cyclopoids (Figure 5, top row; Supporting Information, Figure S10). However, when toxicity is expressed as dissolved Ba, the data points and response curves for Wellington‐2022 cyclopoids overlay the response curve for Somersby cyclopoids (Figure 5, middle row; Supporting Information, Figure S11). This further supports the earlier findings that dissolved Ba, and not precipitated Ba, causes mortality to cyclopoids. It also indicates that the sensitivity of the two cyclopoid species is similar, despite them being different genera from different locations.

### Dissolved Ba toxicity to cyclopoids

The toxicity of dissolved Ba to Wellington‐2022 and Somersby cyclopoids was similar for each test duration (Figure 5 and Table 3; Supporting Information, Figure S11) with 21‐day EC10s of 7.1 (0.72–13) and 6.2 (2.4–10) mg/L, respectively. Low survival in the control treatments and high variability in Ba treatments at the longer exposure duration of 28 days are most likely exacerbating any differences between Wellington‐2022 and Somersby (Supporting Information, Figure S11), along with the unbalanced data sets for Wellington‐2022 (*n* = 41) and Somersby (*n* = 17) used for curve‐fitting. No‐effect concentration values were derived for all exposure times for Wellington‐2022, except for 14 days; however, only the 21‐ and 7‐day NEC curves visually represented the experimental data appropriately (Supporting Information, Figure S12; 21‐day NEC of 3.3 mg/L; Table 3). No‐effect concentration model curve fits could not be applied to Somersby data, and hence, NEC values could not be estimated. Given the similarity in sensitivity of the Wellington‐2022 and Somersby cyclopoids, the two data sets were combined to improve the NEC curve‐fit model. The resulting data set showed a clear threshold or tipping point, with NEC values of 3.2, 3.7, and 26 mg/L for 21, 14, and 7 days, respectively (Figure 5, bottom row, and Table 3; Supporting Information, Figure S13).

**Table 3:** Dissolved Ba toxicity values (milligrams per liter)^a^ for Somersby and Wellington cyclopoids

Exposure (day)	EC50^b^	EC20^b^	EC10^b^	EC5^b^	NEC^c^
Somersby cyclopoids (*Mesocyclops* spp.)					
28	13 (4.7–21)	9.2 (2.8–16)	7.7 (0.1–15)	6.7 (0–15)	
21	**16 (11**–**20)**	**8.5 (4.6**–**12)**	**6.2 (2.4**–**10)**	**4.8 (1.2**–**8.3)**	
14	35 (29–42)	21 (15–27)	16 (10–22)	13 (6.9–19)	
7	56 (36–76)	33 (21–44)	25 (12–38)	20 (6.0–34)	
4	91 (44–139)	43 (29–56)	29 (15–42)	21 (7.4–34)	
2	>80	>80	>80	>80	
Wellington‐2022 cyclopoids (*Diacyclops* sp.)					
28	17 (7.5–26)	4.7 (0.60–8.7)	2.0 (0–4.5)	0.89 (0–2.3)	0.49
21	**35 (23**–**48)**	**13 (5.1**–**22)**	**7.1 (0.72**–**13)**	**3.8 (0**–**8.3)**	**3.3**
14	44 (35–52)	15 (9.2–20)	7.2 (3.3–11)	3.6 (1.1–6.2)	–
7	67 (59–75)	36 (27–44)	23 (15–32)	16 (8.4–23)	21
4	88 (75–100)	47 (34–60)	31 (19–43)	21 (10–32)	103
2	136 (126–145)	88 (75–101)	66 (52–80)	50 (36–65)	141
Combined data set—Somersby and Wellington‐2022 cyclopoids					
28					6.6
21					**3.2**
14					3.7
7					26
4					106
2					697

^a^ Dissolved (<0.45 µm filtered) Ba is calculated as either the time‐weighted average (when dissolved Ba concentration decreased with increasing exposure time) or the 28‐day average (when dissolved Ba concentrations remained stable throughout the toxicity tests) prior to combining data sets. Values in parentheses are 95% confidence limits. Bold values are recommended for use as possible chronic toxicity values.

^b^ Model curve fits for Somersby cyclopoids (all exposures) were log‐normal and for Wellington‐2022 cyclopoids, Weibull Type 1.

^c^ Sigmoidal curve fits were used to derive EC*x* values. No‐effect curve (NEC) fits were also applied to data sets; NECs are preferred when the concentration–response relationship shows a threshold/tipping point. Blanks indicate the NEC fit model could not be applied or the NEC value could not be derived from the NEC fit.

EC*x* = *x*% effect concentration.

## DISCUSSION

### Survival of stygobiont cyclopoids in control treatments

The survival of cyclopoids in controls decreased with increasing test duration, with a significant decrease after 14 days. In published ecotoxicity studies with stygofauna, the response in the control treatment is rarely reported or confirmed (Di Lorenzo et al., 2019). In one study, the control response for cyclopoids collected from Budderoo and Somersby (the same collection site used in the present study) were well within the stated criterion of <10% mortality over 14 to 28 days (Hose et al., 2016). The authors noted that when mortality was observed it typically occurred within 96 h and suggested that mortality may have been due to the handling of organisms before and during test setup rather than to the toxicity test conditions. Control mortality between 96 h and 28 days was a rare occurrence, observed in only one of 15 toxicity tests (Hose et al., 2016). Another study reported <3% mortality for the stygobiont cyclopoid *Parastenocaris germanica* for ≤96‐h test durations (Notenboom et al., 1992). A review by Di Lorenzo et al. (2019) recommends a control response mortality of <20% (i.e., ≥80% survival) for stygofauna species.

For the 28‐day test durations in the present study, control survival was poor, with only one out of 12 toxicity tests achieving <20% mortality. Surprisingly, this was for the toxicity test using synthetic hard water and the only toxicity test to not use a natural groundwater as the test water. Cyclopoids survived just as well in filtered groundwater and in nonnative waters (low‐SO_4_ groundwater and synthetic water) with different water quality characteristics, providing confidence that cyclopoids could be tested in alternative water types if required, allowing for comparisons of taxa across locations where background water quality may differ.

The present study found that survival of stygobiont cyclopoids in control treatments was variable, especially after 14 days, and that further refinement and understanding are needed on the cause of cyclopoid mortality in control treatments before longer test durations can be used reliably and routinely with this taxon. Our use of field‐collected animals means that the test population was likely of mixed ages, despite our limiting test animals to adult stages, and contained both male and female specimens. This variability within the test population may have contributed to the control mortality, and minimizing this variability might improve future tests. Investing in laboratory‐based cultures may also overcome some of these issues and provide an accessible source of animals for toxicity testing. Establishing laboratory cultures of reproducing stygofauna is challenging; however, recent work by Rütz et al. (2023) has generated optimized laboratory culturing protocols for the stygobiont amphipod *Niphargus aquilex*. Further species‐specific adaptations could be applied to establish long‐term cultures of other stygobiont taxa.

### Toxicity of Ba

Sulfate plays a significant role in the precipitation of Ba in water with increasing sulfate concentrations, shifting the concentration–response relationship for total Ba to the right (less toxic). In the present study, the toxicity of Ba to cyclopoids collected from the Wellington site in 2022 could be attributed to dissolved Ba and not precipitated Ba. Dissolved Ba was also the cause of toxicity to cyclopoids from the Somersby site. However, a complete lack of toxicity of precipitated Ba could not be confirmed because Ba precipitates were not a component of the test treatments as a result of the naturally low sulfate concentrations in the test water (1 mg SO_4_/L). Both the Wellington‐2022 and Somersby cyclopoid species had the same sensitivity to dissolved Ba, with 21‐day EC5 values of 3.8 (0–8.3) and 4.8 (1.2–8.3) mg/L and EC10 values of 7.1 (0.72–13) and 6.2 (2.4–10) mg/L, respectively. As expected, the EC5 values were slightly higher than the 21‐day NEC value derived for Wellington‐2022 cyclopoids of 3.3 mg/L; however, more data are needed to derive an NEC value for Somersby cyclopoids.

An NEC of approximately 3 mg/L dissolved Ba was further supported when considering the toxicity data collected in 2021. Despite the very poor survival in control treatments, the concentration–response relationships for total and dissolved Ba in groundwater with 22 mg SO_4_/L followed the expected response (Supporting Information, Figure S14). When normalized to dissolved Ba concentrations, the concentration–response relationships neatly overlaid the Somersby and Wellington‐2022 toxicity data (Supporting Information, Figures S15 and S16) and generated NEC values of 3.1, 3.1, 2.8, 26, 32, and 179 mg/L dissolved Ba for 28, 21, 14, 7, 4, and 2 days, respectively (Supporting Information, Table S12). For this data set, an increase in exposure duration beyond 14 days did not increase dissolved Ba toxicity, a trend that was also observed for the combined Somersby and Wellington‐2022 data set (Table 3). An extended exposure duration did not increase toluene toxicity to a stygobiont amphipod (measured as survival) and was called the “ultimate” toxicity value (Avramov et al., 2013). When this occurs, the toxicity value may be considered to represent chronic (long‐term) toxicity. However, chronic and acute toxicity to groundwater species have not been defined and are likely to differ from those applied for surface‐water taxa because of the unique evolutionary characteristics and traits of stygofauna (Di Lorenzo et al., 2019; Galassi, 2001; Hose, Chariton, et al., 2022; Hose, Dabovic, et al., 2022; Hose et al., 2023).

No other studies have reported Ba toxicity to stygofauna, but data do exist for surface‐water crustaceans. A study with a water flea, *C. dubia*, in synthetic soft water (with and without sulfate) showed that toxicity measured as immobility was also attributed to dissolved Ba and not precipitated Ba, with a 48‐h EC10 of 15 mg Ba/L (Golding et al., 2018). Brix et al. (2010) showed that reproduction in *C. dubia* was a more sensitive test endpoint than adult survival when exposed to BaCO_3_ over 7 days, with a concentration of 10 mg Ba/L causing 25% inhibition in reproduction. The test water had a sulfate concentration of 7.7 mg/L, which suggests that the Ba exposure consisted of dissolved Ba and BaSO_4_ precipitate, although BaCO_3_ precipitate was unlikely at this concentration because it is below the solubility limit. The reproduction of the water flea *Daphnia magna* was also more sensitive than survival based on the 3‐week EC50 of 8.6 mg/L and LC50 of 13.5 mg/L, respectively (Biesinger & Christensen, 1972). A low‐effect concentration of 5.8 mg/L (EC16) was also reported for inhibition of reproduction over 3 weeks. However, it is not known whether the Ba exposure was in the dissolved or precipitated form because the dissolved Ba concentrations were not measured in the toxicity test solutions, nor was the sulfate concentration in the diluent lake water.

The sensitivity of the shrimp *Paratya australiensis* to Ba (96‐h LC20 of 11 mg Ba/L) was similar to that of other crustaceans (Willem et al., 2022). It is difficult to determine if the Ba exposure in that study was in the dissolved or precipitated form because the sulfate concentration in the test water was not measured. It is likely that exposure was predominantly in the dissolved form because the measured dissolved Ba concentrations were >90% of nominal concentrations and no precipitates were visible in test solutions ≤47 mg Ba/L (Willem et al., 2022). Supporting the above toxicity data, 93% survival of *Hyalella azteca* was observed after 7‐day exposure to 1.1 mg/L dissolved Ba (measured) in soft water containing 3.4 mg SO_4_^−^/L (Borgmann et al., 2005).

The chronic toxicity of Ba has also been studied in detail with a freshwater microalga (Golding et al., 2018) and a marine bivalve (Spangenberg & Cherr, 1996). Toxicity to the freshwater microalga *Chlorella* sp., measured as inhibition in cell division rate, was predominantly attributed to precipitated forms of Ba, which was more toxic than dissolved Ba (Golding et al., 2018). The 48‐h EC10 value for exposures of only precipitated BaSO_4_ was 3.5 mg/L, compared to 40 mg/L for dissolved‐only Ba. Barium was highly toxic to early embryo development of *Mytilus californianus*, with effects occurring between 0.2 and 0.9 mg/L total Ba (Spangenberg & Cherr, 1996). All of the Ba was present in the dissolved form up to 0.55 mg/L. Embryonic abnormalities observed included changes in morphology and shell calcification (Spangenberg & Cherr, 1996). Higher concentrations of added Ba were associated with a significant decrease in toxicity and a decrease in dissolved Ba concentrations in seawater to background concentrations (<0.03 mg/L; Spangenberg & Cherr, 1996).

These studies highlight that Ba is similarly, if not more, toxic, to stygobiont cyclopoids than to surface‐water crustaceans and that quantifying Ba speciation and identifying relative ecotoxicological effects are critical when assessing the risk of Ba in natural waters.

### Risk of Ba to groundwater ecosystems

Concentrations of Ba in groundwaters are naturally low (Shand & Edmunds, 2008), but elevated concentrations have been measured in shallow aquifers in Colorado, USA (0.018–0.594 mg/L; Bruce & McMahon, 1996); drinking water supplies in Illinois, USA (1.1–10 mg/L; Calabrese, 1977); and industrial sites associated with oil and gas operations in Texas, USA (0.001–2.3 mg/L; Hudak & Wachal, 2001) and in the Yan'an area in northwest China (0.01–45 mg/L; Zhang et al., 2023). Water quality guidelines for surface waters have been proposed in The Netherlands for dissolved Ba (Verbruggen et al., 2020) using an assessment factor applied to the most sensitive acute and chronic toxicity values. A maximum acceptable concentration environmental quality standard for short‐term concentration peaks of 1.1 mg/L and a chronic quality criterion of 0.62 mg/L were derived.

In the present study, we derived toxicity values for dissolved Ba for two stygobiont cyclopoid species. Based on the minimum data requirements (fewer than three species) and assuming these toxicity values represent chronic toxicity, an assessment factor of 200 applied to the lowest toxicity value (ANZG, 2018; Warne, 2001) would generate guideline values of 0.017 (based on the Wellington‐2022 species NEC of 3.3 mg/L) and 0.024 mg/L (based on the Somersby species EC5 of 4.8 mg/L). These potential guideline values are orders of magnitude lower than those derived by Verbruggen et al. (2020) and highlight several of the challenges to deriving guideline values. Firstly, there are insufficient toxicity data available to meet the minimum requirements of deriving a guideline value using the species sensitivity distribution (SSD) method (Batley et al., 2018; Warne et al., 2018); thus, the resulting values based on the assessment factor method are of unknown reliability. Secondly, the potential guideline values are up to five times lower than the natural concentrations of dissolved Ba in groundwaters used in the present study from which the test species were collected (0.0075–0.085 mg/L; Table 3).

These factors make the potential guideline values derived in the present study implausible to apply to contaminant risk assessments. Despite these uncertainties, the study of groundwaters associated with oil and gas activities in the Yan'an area of northwest China (Zhang et al., 2023) identified concentrations of dissolved Ba in excess of the no‐effect (or low‐effect) toxicity values derived in the present study for the two cyclopoid species, indicating that dissolved Ba poses a risk to groundwater quality. Further toxicity testing with other stygobiont species is recommended to increase the ecotoxicity data available to derive a guideline value for groundwaters using the SSD method.

## CONCLUSION

The present study provides the first data describing Ba toxicity to stygofauna. Toxicity was attributed to dissolved Ba, and not the BaSO_4_ precipitate. Two species of cyclopoid copepods had similar sensitivity to dissolved Ba, with a 21‐day NEC value of 3.3 mg/L for the Wellington species (*Mesocyclops* sp.) and a 21‐day EC5 value of 4.8 mg/L for the Somersby species (*Diacyclops* sp.). Establishing laboratory cultures of stygobiont cyclopoids may address the variable survival of cyclopoids in toxicity tests with longer exposure times (>14 days). Concentrations of dissolved Ba in groundwater exceeding these no‐ and low‐effect concentrations indicate that Ba may pose a risk to groundwater biota. It is recommended that further toxicity testing with other stygobiont species is carried out to meet the requirements for deriving a guideline value for Ba suitable for groundwater contaminant risk assessments.

## Supporting Information

The Supporting Information is available on the Wiley Online Library at https://doi.org/10.1002/etc.5956.

## Conflict of Interest

The authors declare no conflict of interest.

## Author Contributions Statement

**Merrin S. Adams**: Formal analysis; Investigation; Methodology; Writing—original draft; Writing—review & editing. **Kitty S. McKnight, David M. Spadaro, Monique T. Binet**: Formal analysis; Investigation; Methodology; Writing—review & editing. **Grant C. Hose**: Conceptualization; Investigation; Methodology; Resources; Writing—review & editing. **Stephen Fenton**: Conceptualization; Funding acquisition; Project administration; Resources; Writing—review & editing. **Stuart L. Simpson**: Conceptualization; Funding acquisition; Project administration; Supervision; Resources; Writing—review & editing.

## Supporting information

This article includes online‐only Supporting Information.

Dissolved barium causes toxicity to groundwater *Cyclopoida*.

## Data Availability

Data and associated meta data are available from the corresponding author (currently at merrin.adams@environment.nsw.gov.au).
