# Supplementary material for: Dissolved Barium Causes Toxicity to Groundwater Cyclopoida
Source: Environ Toxicol Chem. 2024 Aug 13;43(12):2501–14. doi: 10.1002/etc.5956 (PMC11619739; doi:10.1002/etc.5956)
Supplement: Supplementary file 1 — Dissolved barium causes toxicity to groundwater Cyclopoida. [file ETC-43-2501-s001.docx]

**Dissolved barium causes toxicity to groundwater Cyclopoida**

Merrin S. Adams^a*^, Kitty S. McKnight^a,b^, David M. Spadaro^a^, Monique T. Binet^a^, Grant C. Hose^b^, Stephen Fenton^c^ and Stuart L. Simpson^a,d^

^a^ CSIRO Environment, Lucas Heights, New South Wales 2234, Australia

^b^ School of Natural Sciences, Macquarie University, Macquarie Park, New South Wales 2109, Australia

^c^ Chevron Australia, Perth, Western Australia 6000, Australia

^d^ CSIRO Environment, Dutton Park, Queensland 4102, Australia

* Corresponding author: Merrin Adams (currently at merrin.adams@environment.nsw.gov.au)

**Table S1.** Comparison of the most sensitive ecotoxicity data for metal and toluene toxicity (survival) to stygofauna (based on EC50 and EC10 values summarized in Castaño-Sánchez et al., 2020 and Hose et al., 2019).

| **Toxicant** | **Test Duration** | **Toxicity Value (µg/L)** | **Species Subclass/Order** | **Reference** | **ANZG (2018) Guideline Value (95% protective concentration, µg/L)** |
| --- | --- | --- | --- | --- | --- |
| **EC50 values** | |  |  |  |  |
| As(III) | 28 d  14 d | 250  250 | Copepoda/Cyclopoida  Malacostraca/Syncarida* | Hose et al. (2016)  Hose et al. (2019) | 24 |
| Cu | 48 h | 6,200 | Isopoda | Reboleira et al. (2013) | 0.2 |
| Cd | 96 h | 1,200 | Isopoda | Mösslacher and Notenboom (1999) | 0.2 |
| Cr(VI) | 28 d | 20 | Copepoda/Harpacticoida | Hose et al. (2016) | 1.0 |
| Pb | 48 h | 800 | Amphipoda | Boutin et al. (1995) | 3.4 |
| Ni | 96 h | 76,000 | Isopoda | Barr (1976) | 11 |
| Zn | 48 h | 450 | Amphipoda | Boutin et al. (1995) | 8 |
| Toluene | 14 d | 47,000^a^ | Amphipoda | Avramov et al. (2013) | — |
| **EC10 values** | |  |  |  |  |
| As(III) | 14 d | 10 | Malacostraca/Syncarida* | Hose et al. (2019) | 24 |
| Cr(VI) | 14 d  28 d | 2  2 | Copepoda/Harpacticoida  Copepoda/Harpacticoida | Hose et al. (2016)  Hose et al. (2016) | 1.0 |
| Zn | 28 d | 10 | Copepoda/Copepoida | Hose et al. (2016) | 8 |

^*^ Class/Superorder

^a^ without significant loss of toluene from the toxicity test exposure.

**REFERENCES**

Avramov M., Schmidt SI. & Griebler C. (2013). A new bioassay for the ecotoxicological testing of VOCs on groundwater invertebrates and the effects of toluene on *Niphargus inopinatus*. *Aquatic Toxicology* 130−131, 1−8.

Barr T.C. (1976). Ecological effects of water pollutants in mammoth Cave: Final technical report to the National Park Service. University of Kentucky.

Boutin C., Boulanouar M. & Yacoubi-Khebiza M. (1995). A simple biological test for the lethal toxicity of water and sediment of wells. Compared in vitro toxicity of some heavy metals and ammonium to three genera of aquatic crustacea living in the wells. *Hydroécologie Appliquée* 7 (1−2), 91−109.

Castaño-Sánchez A., Hose G.C. Reboleira A.S.P.S. (2020). Ecotoxicological effects of anthropogenic stressors in subterranean organisms: A review. *Chemosphere* 244:125422.

Hose G.C., Symington K., Lott M. & Lategan M. (2016). The toxicity of arsenic(III), chromium(VI) and zinc to groundwater copepods. *Environmental Science and Pollution Research* 23, 18704−18713.

Hose G.C., Symington K., Lategan M.J. & Siegele R. (2019). The toxicity and uptake of As, Cr and Zn in a stygobitic syncarid (Syncarida: Bathynellidae). *Water* 11, 2508; doi:10.3390/w11122508.

Mösslacher F. & Notenboom J. (1999). Groundwater Biomonitoring. *Environmental Science Forum*, vol. 96. Trans Tech Publications, Switzerland, pp 119−140.

Reboleira A.S.A.P., Abrantes N., Oromí P. & Gonçalves F. (2013). Acute toxicity of copper sulfate and potassium dichromate on stygobiont Proasellus; general aspects of groundwater ecotoxicology and future perspectives. *Water Air Soil Pollution* 224, 1550. [10.1007/s11270-013-1550-0](about:blank)

**Table S2.** Chemical and physico-chemical parameters measured in groundwater and synthetic water.

| **Parameter** | **NMI** | | **CSIRO** | |
| --- | --- | --- | --- | --- |
|  | **Technique** | **Procedure** | **Technique** | **Procedure** |
| total metals and metalloids: Al, As, Ba, B, Cd, Cr, Co, Cu, Fe, Pb, Mn, Mo, Ni, Si, Sr, Ti, V, Zn plus P and S | ICP-AES or ICP-MS^a^ | in-house method NT2_47 | ICP-AES^a^ | C-229: ICP-AES |
| dissolved metals and metalloids (filtered to 0.45 µm): Al, As, Ba, B, Cd, Cr, Co, Cu, Fe, Pb, Mn, Mo, Ni, Si, Sr, Ti, V, Zn plus P and S | ICP-AES or ICP-MS | in-house method NT2_47 | ICP-AES^a^ | C-229: ICP-AES |
| major cations: Ca, Mg, K, Na | ICP-AES | in-house method NT2_47 | ICP-AES^a^ | C-229: ICP-AES |
| major anions: Cl, SO_4_ | Discrete analyser | in-house method NW_D3_B14 for Cl and NW_D10_B14 for SO_4_ | Ion Chromatography | C-261: Anions by Ion Chromatography |
| Nutrients: nitrate and nitrite | Segmented flow analyser | in-house method NWD20 | Ion Chromatography | C-261: Anions by Ion Chromatography |
| Orthophosphate | Discrete analyser | in-house method NWD9 | Discrete Analyser | C-262: Phosphate by Discrete Analyser |
| Total ammonia | Segmented flow analyser | in-house method NWD17 | Discrete Analyser | C-264: Ammonia by Discrete Analyser |
| Dissolved organic carbon (DOC, filtered to 0.45 µm) | Combustion and infrared | in-house method NW_S15 | combustion | C-260: TOC by combustion |
| Total organic carbon (TOC) | Combustion and infrared | in-house method NW_S15 | combustion | C-260: TOC by combustion |
| Alkalinity: bicarbonate, carbonate, and hydroxide (as CaCO_3_) | Titration | in-house method NW_B1 | Titration | C-257: Alkalinity by titration |
| Hardness: Ca, Mg and total (as CaCO_3_) | ICP-AES | in-house method NW_D5 | ICP-AES | C-229: ICP-AES |
| Conductivity | Electrometric | in-house method NW_B9 | Electrometric | Refer to manuscript |
| pH | Electrometric | in-house method NW_S11 | Electrometric | Refer to manuscript |

^a^ ICP-AES, inductively-coupled plasma atomic emission spectrometry; ICP-MS, inductively-coupled plasma mass spectrometry

**Table S3.** Toxicity test conditions for stygobiont cyclopoids.

| Parameter | Result |
| --- | --- |
| Test type | Static |
| Temperature | 18 ± 1°C |
| Light | None (except for short exposures during observations under the microscope). |
| Test container | 24-well plates, untreated |
| Test vessel volume | 2.5 mL |
| Test solution volume | 2 mL |
| Renewal of test solutions | None |
| Age of test organisms | Unknown age or life stage (tests were initiated within 16 days of collection). |
| No. of animals per well | One^1^ |
| No. of replicate wells per concentration/treatment | Variable, depending on the number of isolated animals.  Generally aiming for at least 10 replicates per treatment (based on 1 animal per well). |
| No. of concentrations/treatments | Variable, depending on the number of isolates animals.  For concentration-response relationships, generally aiming for 7 plus a control. |
| Dilution water | A. Groundwater from the test species’ collection site, 63 µm-filtered at the time of collection and decanted after solids settled out of suspension.  B. Low-SO_4_ groundwater, decanted after solids settled out of suspension.  C. Synthetic water (sulfate-free, 0.45 µm filtered). |
| Test duration | 28 d, with time points including 2, 4, 7, 14, 21 d |
| Endpoint | Survival. Survival was defined as any movement of body or sensory appendages within 15 s of being gently prodded when viewed under a microscope. Assessments of survival occurred at all time points and all replicates, even those previously classified as dead because in some instances organisms were initially misclassified due to their inherent lack of movement. This provided certainty that mortality rather than dormancy/immobility (Hose et al., 2016) was measured. |
| Test acceptability | Control mortality <20% (Di Lorenzo et al., 2019). Higher mortality in control treatments may be observed during the method development phase, especially for long exposure durations. |

^1^ Occasionally two animals were transferred to an individual test well. In this case, the two animals were left in the well and survival of each recorded over 28 days. The removal of one of the animals from the test well was considered to potentially cause undue stress on both animals.

**Table S4.** Composition of groundwaters and synthetic hardwater used in toxicity tests.

| **Site** | **Somersby** | | **Wellington** | | **Wellington** | | **Wellington** | | **Low-SO_4_** | | **Synthetic water** | |
| --- | --- | --- | --- | --- | --- | --- | --- | --- | --- | --- | --- | --- |
| **Bore** | **75039** | | **WRS-05** | | **WRS-05** | | **WRS-05** | |  | |  | |
| **Collection date** | **2020** | | **2021** | | **Apr, 2022** | | **Aug, 2022** | | **May, 2022** | | **May-22** | |
|  | **Filtered** | **Total^^^** | **Filtered** | **Total^^^** | **Filtered** | **Total^^^** | **Filtered** | **Total^^^** | **Filtered** | **Total^^^** | **Filtered** | **Total^^^** |
| ***Metals (µg/L)*** |  |  |  |  |  |  |  |  |  |  |  |  |
| Al | 452 | 2470 | 0.7 | 236 | <5 | 18 | <5 | 2500 | <5 | 1300 | <5 | <5 |
| As | <1 | <1 | 3 | 2 | <1 | <1 | <1 | 1.7 | <1 | 4.3 | <1 | <1 |
| B | 15 | 15 | 37 | 35 | 37 | 37 | 24 | 25 | 88 | 90 | <5 | <5 |
| Ba | 7.5 | 15 | 16 | 17 | 85 | 94 | 80 | 98 | 41 | 47 | <1 | <1 |
| Cd | <1 | <1 | <1 | <1 | <0.1 | <0.1 | <0.1 | <0.1 | <0.1 | <0.1 | <0.1 | <0.1 |
| Co | <1 | 1 | <1 | <1 | <1 | <1 | <1 | 3.7 | <1 | 1.9 | <1 | <1 |
| Cr | <1 | 2 | <1 | <1 | <1 | <1 | <1 | 5.8 | <1 | 7.8 | <1 | <1 |
| Cu | 2 | 3 | 2 | 3 | <1 | <1 | <1 | 5.7 | <1 | 3.8 | <1 | <1 |
| Fe | 3 | 478 | <1 | 252 | <5 | 29 | <5 | 4100 | <5 | 6800 | <5 | <5 |
| Mn | 7.6 | 8.2 | <0.1 | 12 | <1 | <1 | <1 | 700 | <1 | 100 | <1 | <1 |
| Mo | <1 | <1 | <1 | <1 | <1 | <1 | <1 | <1 | <1 | <1 | <1 | <1 |
| Ni | 1 | 2 | 4 | 5 | <1 | <1 | <1 | 3.6 | <1 | 2.4 | 1.7 | 1.7 |
| P | 8 | 21 | 18 | 24 | 50 | 71 | 69 | 130 | <50 | <50 | <50 | <50 |
| Pb | <2 | <2 | <2 | <2 | <1 | <1 | <1 | 2 | <1 | 1.2 | <1 | <1 |
| Sr | 11 | 16 | 726 | 725 | 710 | 770 | 760 | 760 | 200 | 200 | <1 | <1 |
| Ti | <1 | 102 | <1 | 8 | <5 | <5 | <5 | 70 | <5 | 97 | <5 | <5 |
| V | <1 | 2 | 7 | 8 | 2.1 | 2 | 2.2 | 15 | <1 | 16 | <1 | <1 |
| Zn | 13 | 13 | 7 | 8 | <1 | <1 | <1 | 9.6 | 1.3 | 11 | <1 | <1 |
| ***Major ions (mg/L)*** |  |  |  |  |  |  |  |  |  |  |  |  |
| Ca | 0.5 | 0.5 | 100 | 100 | 83 | 94 | 85 | 88 | 130 | 130 | 64 | 65 |
| K | 0.9 | 1.1 | 2.4 | 2.4 | 1.6 | 1.6 | 1.0 | 3.2 | 1.4 | 1.4 | 10 | 9.6 |
| Mg | 5 | 5.1 | 48 | 48 | 42 | 45 | 44 | 43 | 4.3 | 4.3 | 62 | 58 |
| Na | 13 | 13 | 39 | 39 | 76 | 80 | 70 | 72 | 40 | 39 | 130 | 120 |
| S | 0.5 | 0.5 | 8 | 8 | 41 | 43 | 35 | 34 | 3.8 | 3.7 | <0.05 | <0.05 |
| Si | 3.8 | 6.7 | 18 | 18 | 14 | 15 | 13 | 18 | 6.6 | 8.8 | <0.05 | <0.05 |
| Chloride | 28 | --- | 50 | --- | 76 | --- | 82 | --- | 48 | --- | 300 | --- |
| Sulfate | 1 | --- | 22 | --- | 89 | --- | 110 | --- | 7.4 | --- | <0.1 | --- |
| ***Nutrients/Other*** |  |  |  |  |  |  |  |  |  |  |  |  |
| PO_4_-P (µg/L) | <10 | --- | <10 | --- | 40 | --- | 34 | --- | 8 | --- | <5 | --- |
| NO_3_-N (mg/L) | 3.3 | --- | 1.8 | --- | 0.1 | --- | 0.11 | --- | 0.85 | --- | 0.028 | --- |
| NO_2_-N (mg/L) | <0.04 | --- | <0.04 | --- | <0.005 | --- | <0.005 | --- | <0.005 | --- | <0.005 | --- |
| Total ammonia-N (µg/L) | 226 | --- | 95 | --- | 4 | --- | 27 | --- | 22 | --- | 19 | --- |
| DOC (mg/L) | <1 | --- | 2 | --- | 2.4 | --- | 1.9 | --- | 0.8 | --- | <0.5 | --- |
| TOC (mg/L) | --- | <1 | --- | 2 | --- | 2.2 | --- | 2.0 | --- | 1.5 | --- | <0.5 |
| Alkalinity^a^ (mg/L CaCO_3_) | --- | <10 | --- | 370 | --- | 300 | --- | 310 | --- | 320 | --- | 240 |
| Hardness^b^ (mg/L CaCO_3_) | 22 | 22 | 447 | 445 | 428 | 431 | --- | 424 | --- | 308 | --- | 437 |
| pH | --- | 4.6 | --- | 4.7 | --- | 7.7 | --- | 7.4 | --- | 7.55 | --- | 7.9 |
| Conductivity (µS/cm) | --- | 150 | --- | 990 | --- | 1090 | --- | 1100 | --- | 783 | --- | 1500 |
| Salinity (PSU) | --- | 0.7 | --- | --- | --- | 0.54 | --- | 0.4 | --- | --- | --- | 0.69 |

^ Measured total metal concentrations may not represent the homogenised groundwater samples because in some cases the groundwater was left to settle prior to sub-sampling for analysis to better reflect the concentrations in toxicity test solutions.

^a^ Alkalinity reported as bicarbonate (as CaCO_3_), alkalinity as carbonate and hydroxide were <5 mg CaCO_3_/L respectively

^b^ Hardness reported as the sum of hardness as calcium (as CaCO_3_) and hardness as magnesium (as CaCO_3_)

**Table S5.** Survival of stygobiont cyclopoids from Wellington (2022 and 2021) and Somersby (2020) in native groundwater, Low-SO_4_ groundwater and sulfate-free synthetic water.

| **Test ID** | **Taxa** | | | **Test Water** | **Culture** | **Survival, % Survival** | | | | | | **Animals** |
| --- | --- | --- | --- | --- | --- | --- | --- | --- | --- | --- | --- | --- |
|  | **Isolate** | **Coll#** | **Test#** |  | **Age (d)** | **2 d** | **4 d** | **7 d** | **14 d** | **21 d** | **28 d** | **(n)** |
| 2020 | Somersby Cyclopoids | 2 | 10 | Somersby-2020 | 18 | 100 | 100 | 100 | 100 | 82 | 36 | 11 |
| 2020 | Somersby Cyclopoids | 2 | 14 | Somersby-2020 | 35 | 100 | 100 | 100 | 100 | 100 | 71 | 7 |
| 2021 | Wellington Cyclopoids | 1 | 1 | Wellington-2021 | 3 | 100 | 50 | 50 | 50 | 50 | 38 | 8 |
| 2021 | Wellington Cyclopoids | 1 | 2 | Wellington-2021 | 10 | 74 | 58 | 58 | 55 | 45 | 32 | 31 |
| 50 | Wellington Cyclopoids | 1 | 1 | Wellington-2022, Coll#1 | 8 | 100 | 100 | 100 | 90 | 90 | 60 | 10 |
| 55 | Wellington Cyclopoids | 1 | 2 | Wellington-2022, Coll#1 | 12 | 95 | 92 | 84 | 76 | 46 | 38 | 37 |
| 69 | Wellington Cyclopoids | 2 | 1 | Low-SO_4_ | 4 | 100 | 100 | 100 | 84 | 79 | 63 | 19 |
| 75 | Wellington Cyclopoids | 2 | 2 | Low-SO_4_ | 10 | 100 | 100 | 100 | 100 | 75 | 75 | 12 |
| 65 | Wellington Cyclopoids | 2 | 1 | W1 - Wellington-2022, Coll#2 | 4 | 100 | 100 | 93 | 93 | 86 | 71 | 14 |
| 66 | Wellington Cyclopoids | 2 | 1 | W2 - Wellington-2022, Coll#2 Filtered | 4 | 100 | 93 | 93 | 93 | 86 | 71 | 14 |
| 67 | Wellington Cyclopoids | 2 | 1 | W3 - Wellington-2022, Coll#2 Ba-equilibrated and filtered | 4 | 93 | 93 | 93 | 93 | 80 | 53 | 15 |
| 68 | Wellington Cyclopoids | 2 | 1 | W4 - Sulfate-free synthetic water | 4 | 100 | 100 | 100 | 100 | 100 | 86 | 14 |

Treatment (added barium)

Treatment (added barium)

Treatment (added barium)


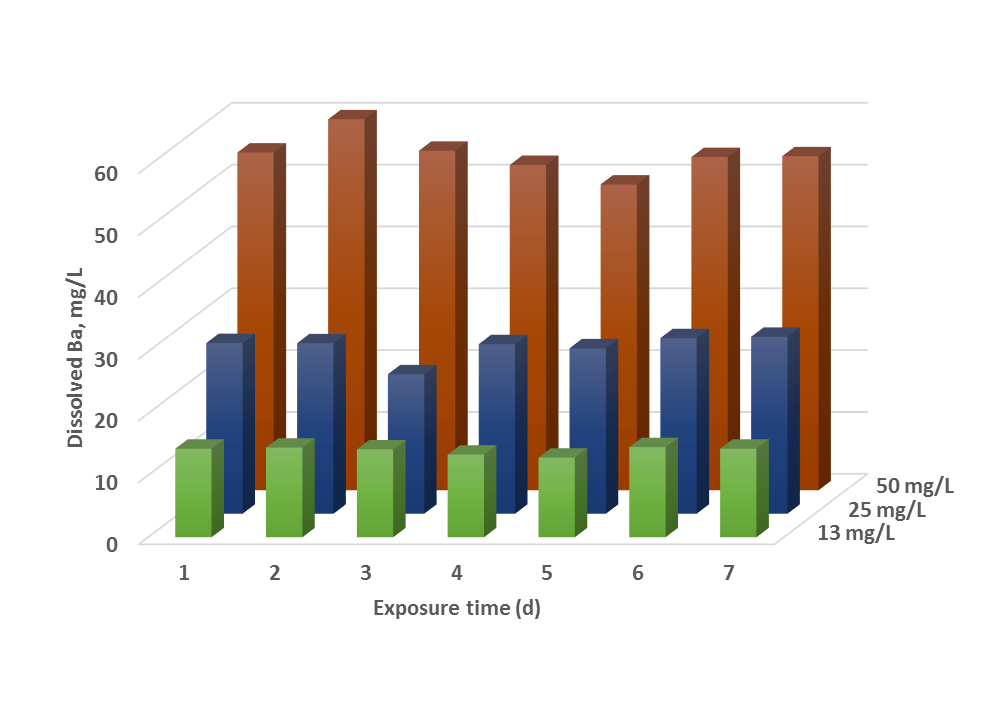

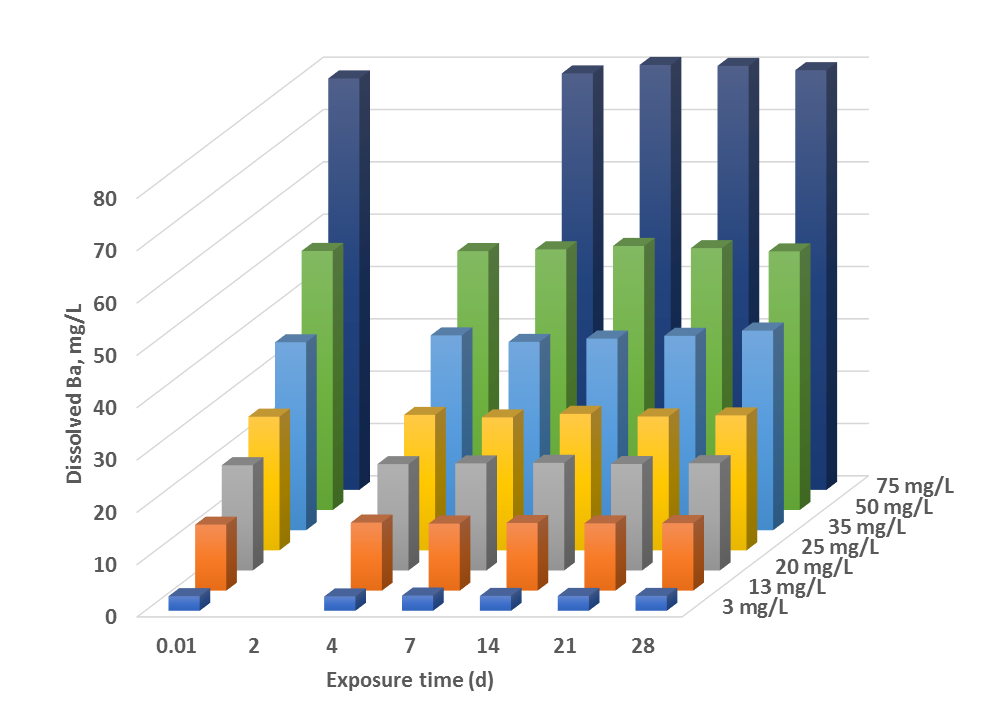

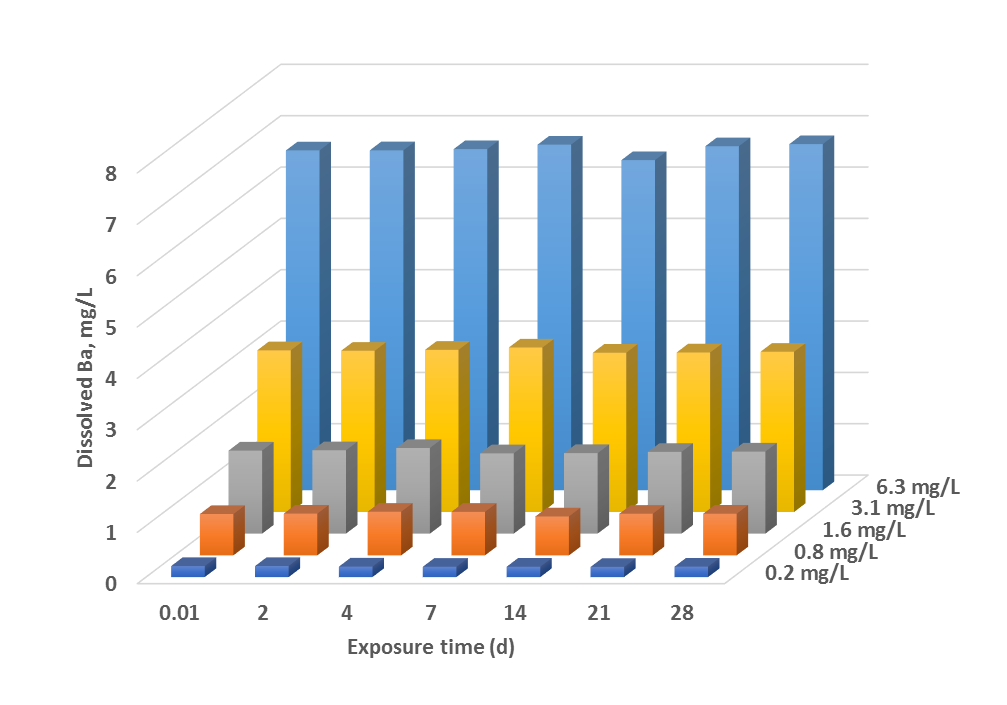


**A**

**B**

**C**

**Figure S1.** Concentrations of dissolved (<0.45 µm filtered) barium over 28-d in Somersby groundwater (2020 Collection #2) from bore 75039 (1 mg/L sulfate). A) and B) are data from toxicity test 10. C) is data from toxicity test 14. The z-axis describes the treatment concentration as the nominal added barium. Blanks indicate the sample/timepoint was not measured.

Treatment (added barium)

Treatment (added barium)


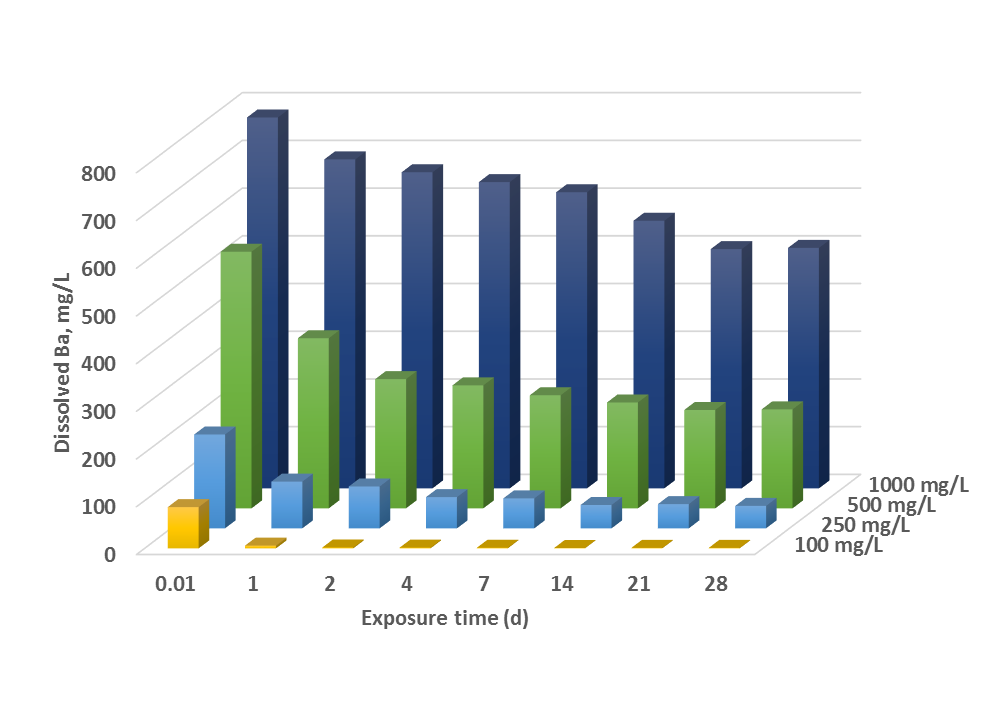

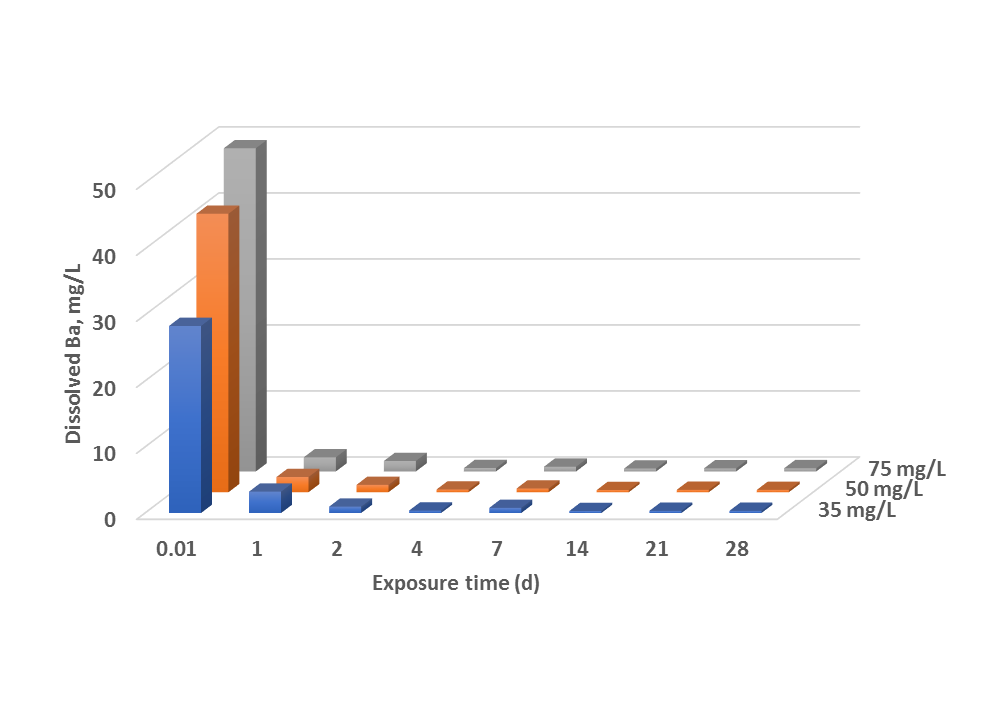


**B**

**A**

**Figure S2.** Concentration of dissolved (<0.45 µm filtered) barium over 28-d in Wellington groundwater (2021 Collection) from bore WRS05 (22 mg SO_4_/L, re-analysis in 2022 measured 65 mg SO_4_/L). A) and B) shows data from a re-simulated toxicity test scenario in 2022. The z-axis describes the treatment concentration as the nominal added barium.


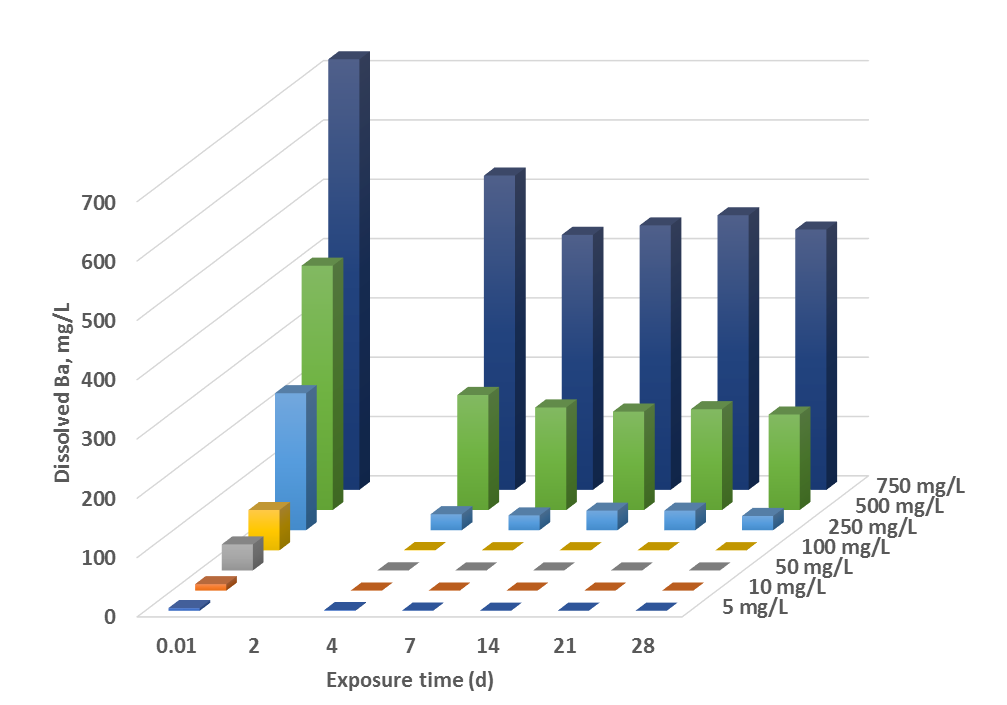

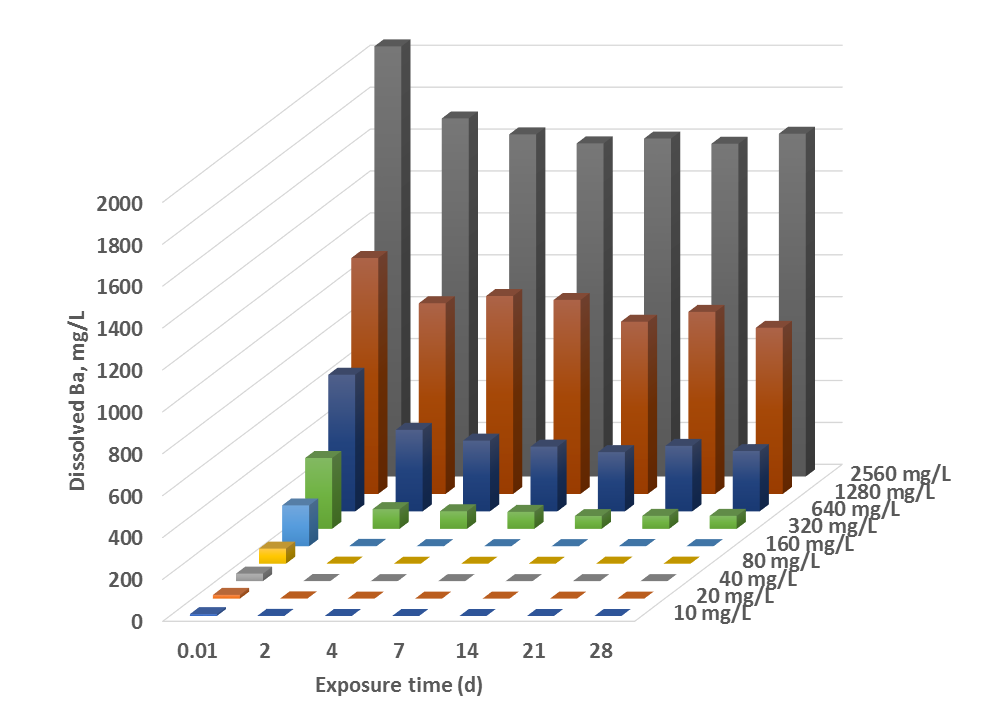

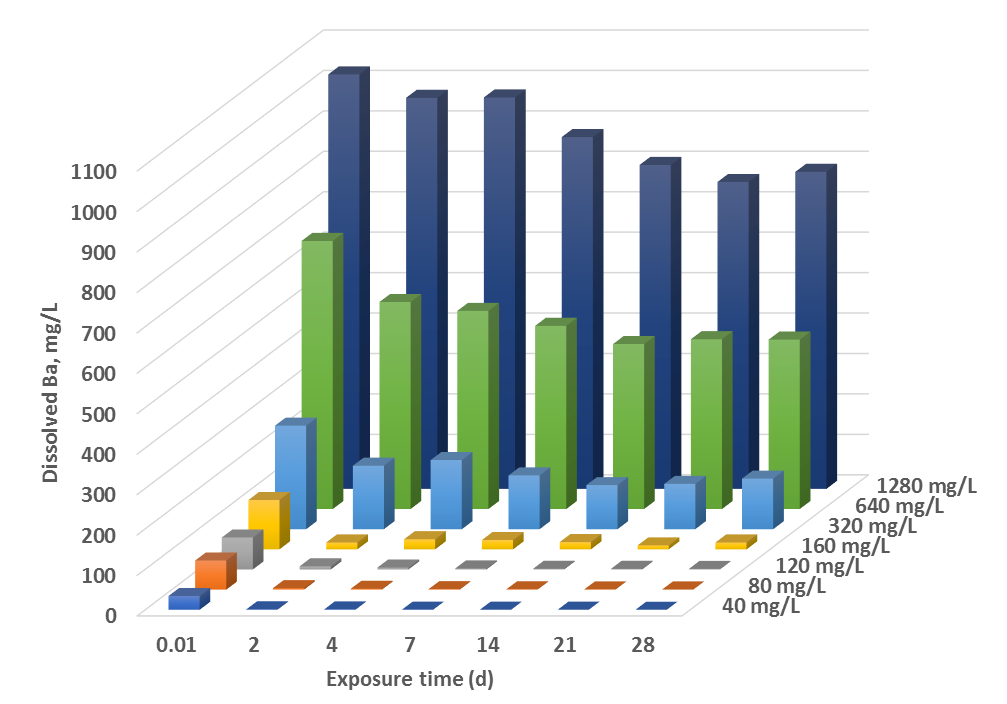

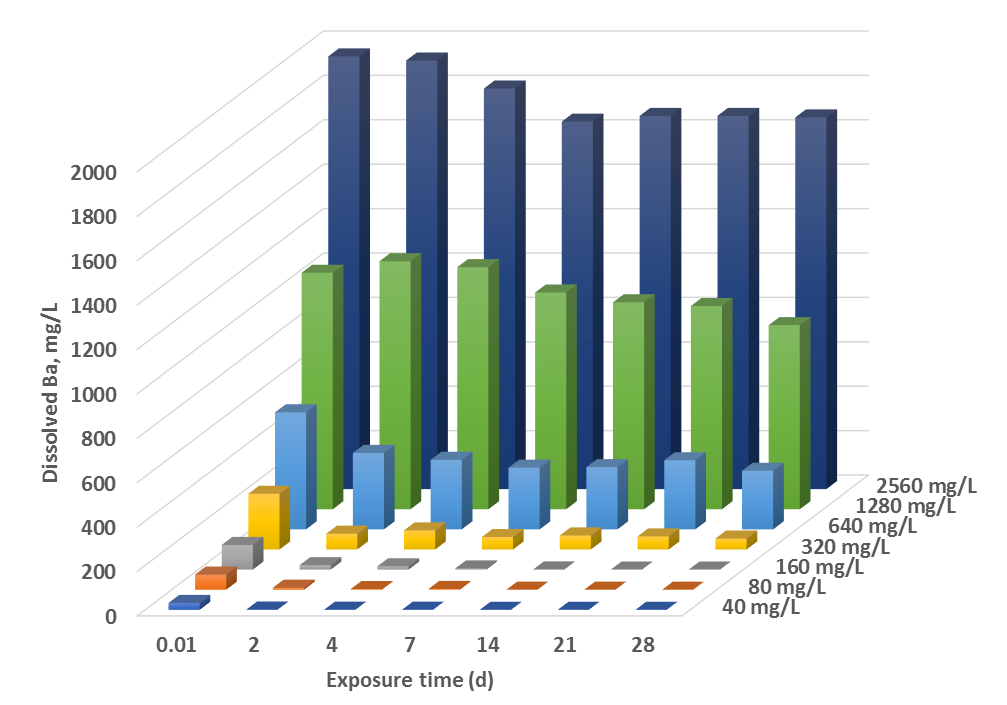


**A**

**B**

**D**

**C**

**Figure S3.** Concentration of dissolved (<0.45 µm filtered) barium over 28-d in Wellington groundwater collected in 2022. Groundwater from Collection #1 (89 mg SO_4_/L) for (A) Test#1 (B) Test#2 (C) and Test#3, and, Collection #2 (110 mgSO_4_/L) (D). The z-axis describes the treatment concentration as the nominal added barium. Blanks indicate the sample/timepoint was not measured. Note the different treatments, colours and y-axis scale in each plot.

Treatment (added barium)

Treatment (added barium)

Treatment (added barium)

Treatment (added barium)


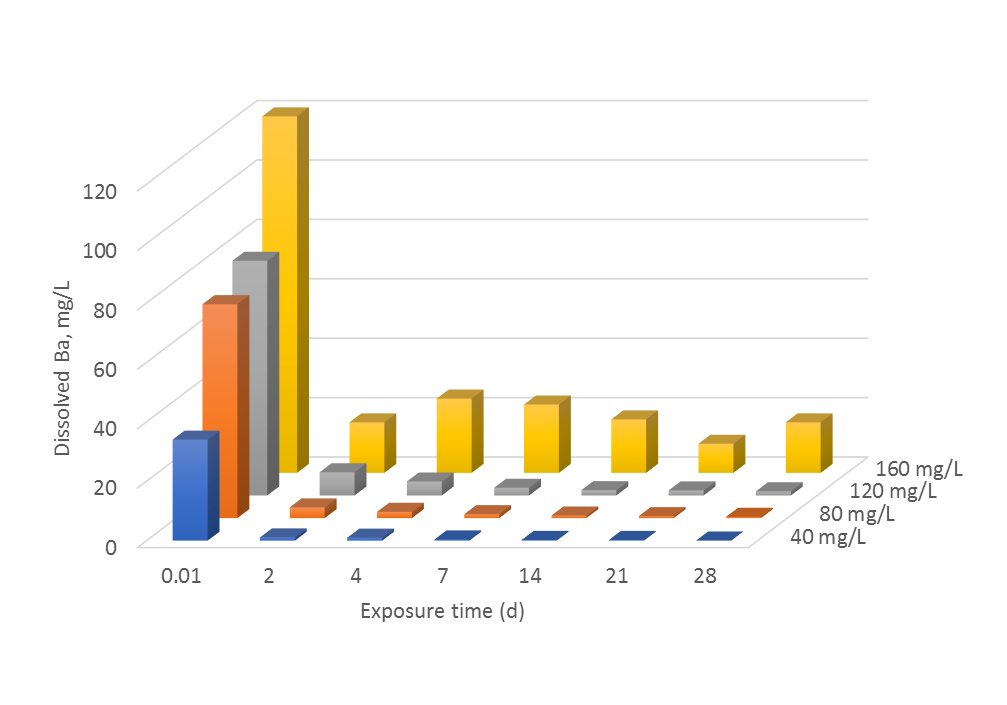

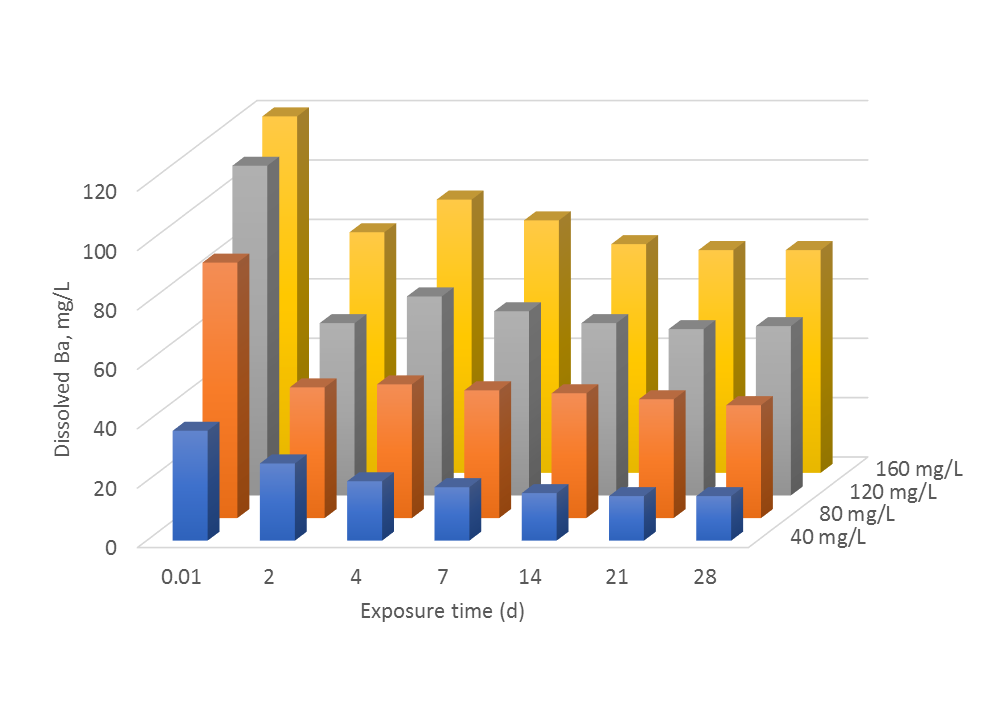

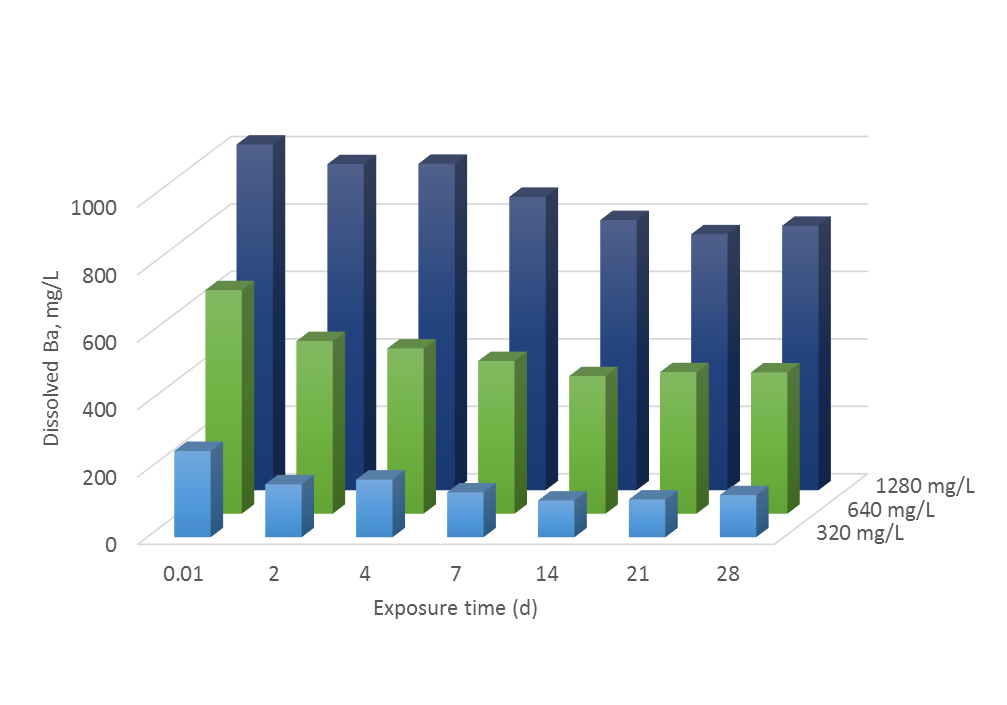

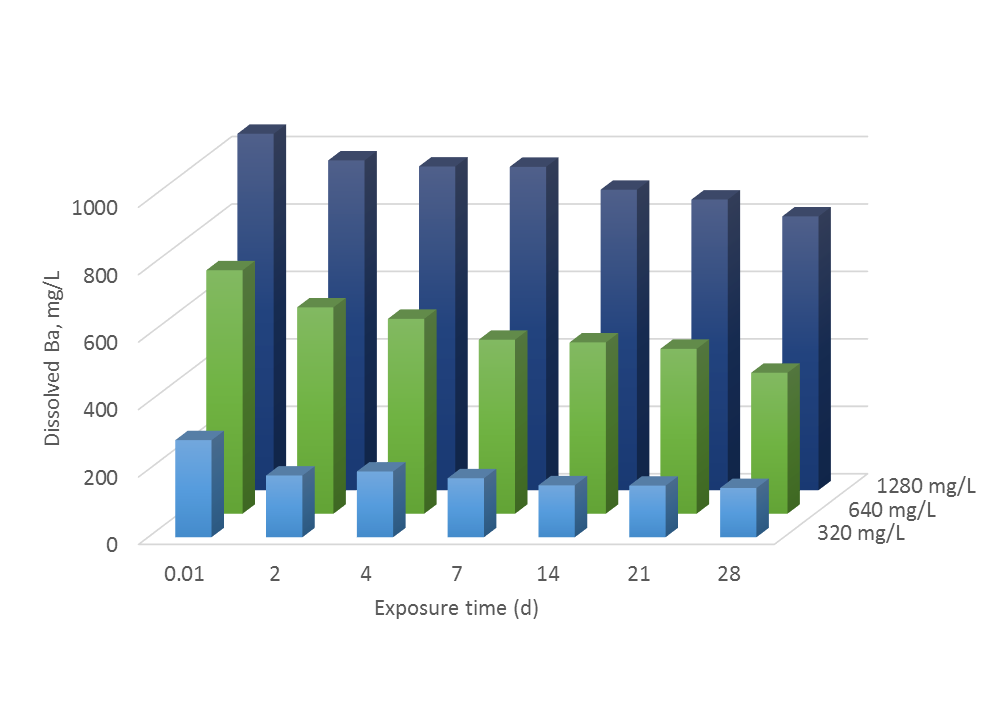


**A**

**C**

**D**

**B**

**Figure S4.** Concentration of dissolved (<0.45 µm filtered) barium over 28-d in Wellington groundwater collected in 2022 Collection #2 (110 mg SO_4_/L) (A, C), compared to a low-sulfate groundwater (7 mg SO_4_/L) (B, D). The z-axis describes the treatment barium concentration as the nominal added barium.

Treatment (added barium)

Treatment (added barium)

Treatment (added barium)

Treatment (added barium)

**W1**

**W2**

**W3**

**W4**


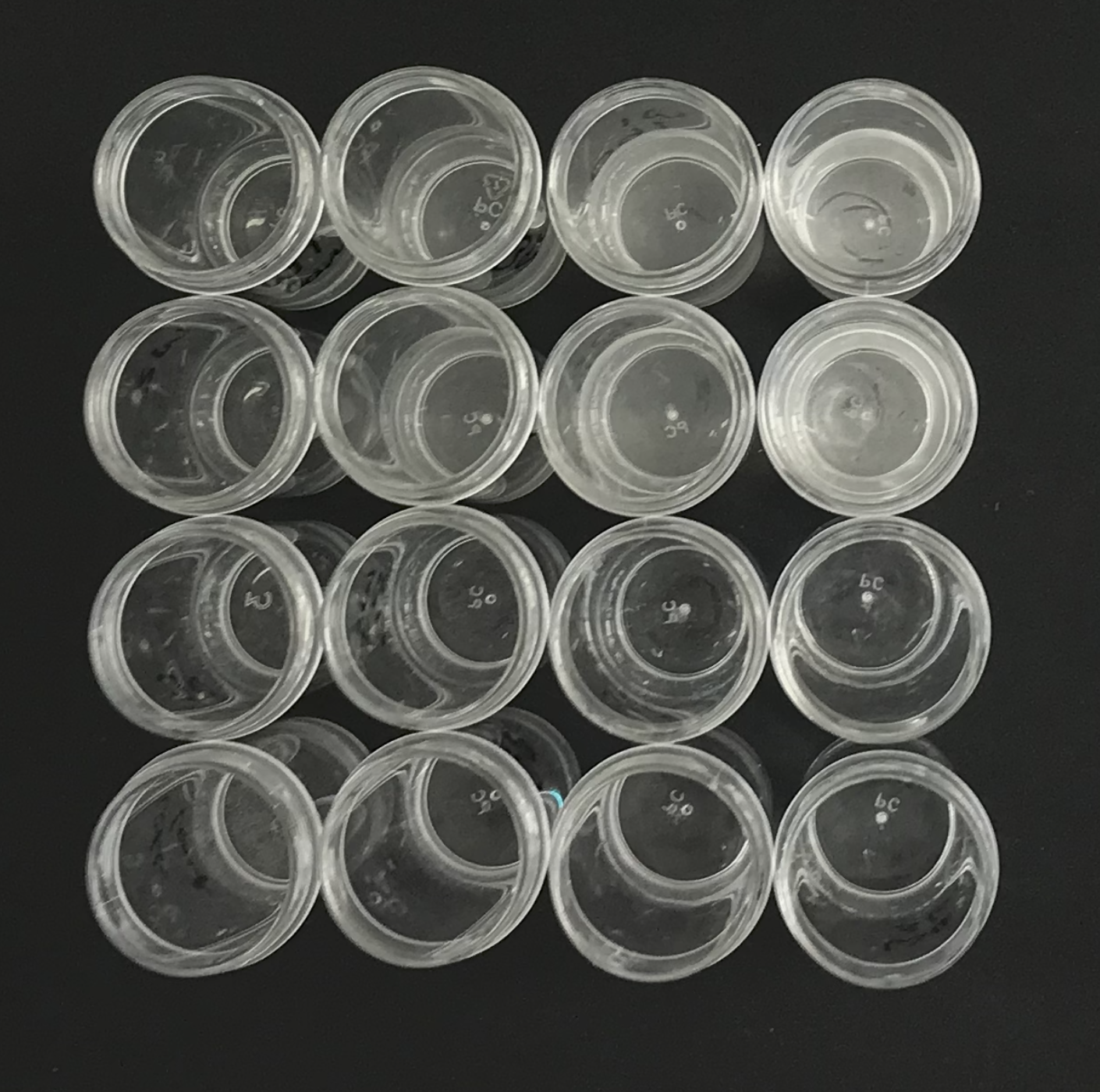


**Control 80 mg Ba/L 160 mg Ba/L 320 mg Ba/L**

**Figure S5.** Photo of simulated toxicity test waters: W1, W2, W3 and W4 with increasing concentrations of added dissolved barium (as barium chloride) and showing the white barium sulfate precipitate (and possibly barium carbonate) in W1 and W2. W1 = Wellington-2022 Coll#2 groundwater following decanting to remove particulates; W2 = as for W1 plus filtration to 0.45 µm; W3 = as for W1 and after equilibration at 18°C for 88 h plus filtration to 0.45 µm; W4 = sulfate-free synthetic water.

**Table S6.** Summary of barium toxicity to Somersby cyclopoids (survival) in native groundwater (1 mg/L sulfate)

**Table S7.** Summary of barium toxicity to Wellington cyclopoids (survival) collected in 2021 and tested in native groundwater (22 mg/L sulfate*)

* Re-analysis measured 65 mg SO_4_/L

**Table S8.** Summary of barium toxicity to Wellington cyclopoids (survival) collected in 2022 and tested in native groundwater (89 and 110 mg/L sulfate for collection #1 and #2, respectively) and Low-SO_4_ groundwater (7 mg/L sulfate)

**Table S9.** Summary of barium toxicity to Wellington cyclopoids (survival) collected in 2022 and tested in different water types^a^

^a^ Wellington groundwater prepared with (W1, W2) and without (W3, W4) barium precipitate

W1 = Wellington groundwater with BaCl2 spike added (as pre previous toxicity tests, dissolved and precipitated Ba)

W2 = Wellington groundwater, filtered to 0.45 µm, then spiked with BaCl2 (i.e. dissolved and precipitated Ba, no food, no GW particulates)

W3 = Wellington groundwater spiked with BaCl_2_, incubate at 18°C dark (stygo cabinet) for at least 88 h (ideally 96 h), filter to 0.45 µm (i.e. dissolved Ba only, no food, no GW particulates, no Ba precipitates)

W4 = synthetic hardwater (430 mg/L CaCO_3_ hardness, pH ~7.1) filtered (i.e. dissolved Ba only, no sulfate in water)

**Figure S6.** Survival of cyclopoids collected from Wellington (2022, Collection #2) as a percentage of the proportion surviving in different waters. Exposure concentrations are expressed as time-weighted average (TWA) for dissolved and precipitated barium exposures (open and partially open data points) or, 28-d average dissolved (<0.45 µm filtered) barium concentrations for dissolved-only barium exposures (solid data points). Exposures were prepared by adding dissolved BaCl_2_ to Wellington (2022, Collection #2) groundwater with 110 mg SO_4_/L following decanting to remove particulates (W1 (open triangle): dissolved and precipitated barium) and after filtration to 0.45 µm (W2 (open diamond): dissolved and precipitated barium). Groundwater with a dissolved-only barium exposure was prepared by adding dissolved BaCl_2_ to Wellington groundwater, equilibrated at 18°C for 88 h then filtered to 0.45 µm (W3 (solid square): dissolved-only barium). Dissolved BaCl_2_ was also added to sulfate-free synthetic hardwater (W4 (solid triangle): dissolved-only barium). Groundwater with a low sulfate concentration (7 mg SO_4_/L) was used to prepare a dissolved and precipitated barium exposure with a lower proportion of insoluble precipitated barium particles (LowSO4 (open square with cross)). The model curve fit for each exposure duration represents the best fit (based on AIC and visual observation) when all data points are combined.

**Table S10.** Analysis of variance (ANOVA) for modelled concentration-response curves of Wellington cyclopoid survival and dissolved (<0.45 µm filtered) barium concentrations with and without precipitated barium*

| **Combined data* model** | **Data* removed** | **Exposure (d)** | **Model** | **F value** | **p value** | **Significant difference^ (p<0.05)** |
| --- | --- | --- | --- | --- | --- | --- |
| W1, W2, W3, W4, LowSO_4_ | W3, W4 | 28 | Weibull, type 1.3 (upper = 100) | 1.3530 | 0.3015 | No |
| (All data) | (Dissolved Ba only) | 21 | Weibull, type 1.3 (upper = 100) | 1.6889 | 0.1926 | No |
|  |  | 14 | Weibull, type 1.3 (upper = 100)) | 2.7526 | 0.0508 | No |
|  |  | 7 | Weibull, type 1.3 (upper = 100) | 1.4656 | 0.2594 | No |
|  |  | 4 | Weibull, type 1.3 (upper = 100) | 1.5750 | 0.2241 | No |
|  |  | 2 | Weibull, type 1.3 (upper = 100) | 0.4157 | 0.8917 | No |

* Barium exposures in test waters;

- W1 & W2 = dissolved and precipitated barium (110 mg/L SO_4_)
- W3 & W4 = dissolved barium only
- Low-SO_4_ = predominately dissolved barium, small amount of precipitated barium (7 mg SO_4_/L)

^All 28-d control survival was very low, and lower than acceptable criteria, indicating tests for significant differences may be unreliable for 28-d data.

**Table S11.** Dissolved (<0.45 µm filtered) barium* toxicity values for Wellington cyclopoids (collected in 2022 Collection #2)

| **Exposure (d)** | **Toxicity value, mg/L, dissolved barium*** | | | | **Curve-fit model^** |
| --- | --- | --- | --- | --- | --- |
|  | **EC50** | **EC20** | **EC10** | **EC5** |  |
| 28 | 12 (6.0-17) | 4.0 (0.93-7.0) | 2.5 (0.17-4.8) | 1.8 (0-3.7) | Weibull, type 2 |
| 21 | 34 (24-44) | 9.2 (3.5-15) | 3.9 (0.39-7.4) | 1.7 (0-3.7) | Weibull, type 1 |
| 14 | 42 (32-53) | 12 (5.3-19) | 5.3 (0.99-9.7) | 2.4 (0-5.0) | Weibull, type 1 |
| 7 | 66 (54-78) | 32 (19-44) | 19 (8.0-31) | 12 (2.7-22) | Weibull, type 1 |
| 4 | 84 (70-98) | 44 (28-59) | 28 (14-43) | 18 (5.8-31) | Weibull, type 1 |
| 2 | 133 (120-145) | 81 (64-99) | 59 (41-77) | 43 (26-61) | Weibull, type 1 |

* Dissolved barium is calculated as either the time-weighted-average (when dissolved barium concentration decreased with increasing exposure time) or the 28-d average (when dissolved barium concentrations remined stable throughout the toxicity tests) prior to combing data sets. Values in parentheses are 95% confidence limits.

^ Sigmoidal curve fits were applied to the combined data sets. The no-effect curve (NEC) curve fit could not be applied to these data sets (lack of a threshold or tipping point).

**Figure S7.** Concentration-response relationships for Somersby cyclopoids expressed as dissolved (<0.45 µm filtered) and total barium in Somersby groundwater (1 mg SO_4_/L). Data from two toxicity test are combined with the best-fit curve models shown (log-normal for 28-, 21- and 14-d; Weibull Type 2 for 7- and 4-d). Dissolved barium concentrations for all exposure durations are reported as the 28-d average of dissolved (0.45 µm) measured barium concentration for each treatment.

**Figure S8.** Concentration-response relationships for Wellington cyclopoids expressed as dissolved (<0.45 µm filtered) and total barium in Low-SO_4_ groundwater (7 mg SO_4_/L). The best-fit curve models are shown for each data set (dissolved barium = Weibull Type 1 for 4-, 7-, 14- and 28-d, Log-logistic for 2-d, log-normal for 21-d; total barium = Weibull Type 1 for 2- to 14-d and 28-d, log-logistic for 21-d). Dissolved barium concentrations are reported as the time-weighted-average (TWA) for each individual exposure duration.

**Figure S9.** Concentration-response relationships for Wellington cyclopoids expressed as dissolved (<0.45 µm filtered) and total barium in Wellington groundwater (average 100 mg SO_4_/L). Data from two collections of Wellington cyclopoids tested in native groundwater are combined with the best-fit curve models are shown (dissolved barium = Weibull Type 1 for 2- to 28-d; total barium = Weibull Type 1 for 28-d and log-normal for 2- to 21-d). Dissolved barium concentrations are reported as the time-weighted-average (TWA) for each individual exposure duration.

**Figure S10.** Concentration-response curve for Cyclopoida (survival) exposure to barium, as total (nominal, added). Data for cyclopoids collected from Wellington (2022 Collection #1 = solid square; 2022 Collection #2 solid triangle), and Somersby (open triangle) for all water types test. Lines show the log-normal curve fits. For 2-d plot, data points are not shown for Somersby cyclopoids where 100% survival was observed for all concentrations tested up to 80 mg Ba/L****

**Figure S11.** Concentration-response curve for Cyclopoida (survival) exposure to barium, as dissolved (<0.45 µm filtered) barium. Data for cyclopoids collected from Wellington (2022 Collection #1 = solid square; 2022 Collection #2 solid triangle), and Somersby (open triangle) for all water types test. Solid line indicates curve fit for Wellington (Weibull type 1, solid) and Somersby (log-normal, dashed) cyclopoids. For 2-d plot, data points are not shown for Somersby cyclopoids where 100% survival was observed for all concentrations tested up to 80 mg Ba/L.

**Figure S12.** Concentration-response curve for Cyclopoida (survival) exposure to barium, as dissolved (<0.45 µm filtered) barium. Modelled No-effect (NEC) curve includes all data sets generated for cyclopoids collected from Wellington in 2022 (Collection #1 = solid square; Collection #2 solid triangle) for all water types test. Dashed line indicates NEC value. Note that a NEC value could not be extrapolated for a 14-d exposure. A sigmoidal curve fit and EC5 or EC10 value should be used for this test durations.

**Figure S13.** Concentration-response curve for Cyclopoida (survival) exposure to barium, as dissolved (<0.45 µm filtered) barium. Modelled No-effect (NEC) curve includes all data sets generated for cyclopoids collected from Wellington (2022 Collection #1 = solid square; 2022 Collection #2 solid triangle), and Somersby (open triangle) for all water types test. Dashed line indicates NEC value. For 2-d plot, data points are not shown for Somersby cyclopoids where 100% survival was observed for all concentrations tested up to 80 mg Ba/L (note that Somersby data points were incorporated into the NEC modelled curve fit).

**Figure S14.** Concentration-response relationships for Wellington cyclopoids expressed as dissolved (<0.45 µm filtered) and total barium in Wellington groundwater (22 mg SO_4_/L, re-analysis in 2022 measured 65 mg SO_4_/L). Data from two toxicity test are combined with the best-fit curve models are shown (dissolved barium = Weibull Type 1 for 2- to 28-d; total barium = Weibull Type 1 for 28-d and log-normal for 2- to 21-d). Dissolved barium concentrations are reported as the time-weighted-average (TWA) for each individual exposure duration.

**Figure S15.** Concentration-response curve for Cyclopoida (survival) exposure to barium, as total (nominal added). Data for cyclopoids collected from Wellington (2022 Collection #1 = solid square; 2022 Collection #2 solid triangle, 2021 = solid circle) and Somersby (open triangle) for all water types test. For 2-d plot, data points are not shown for Somersby cyclopoids where 100% survival was observed for all concentrations tested up to 80 mg Ba/L.

**Figure S16.** Concentration-response curve for Cyclopoida (survival) exposure to barium, as dissolved (<0.45 µm filtered) barium. Modelled No-effect (NEC) curve includes all data sets generated for cyclopoids collected from Wellington (2022 Collection #1 = solid square; 2022 Collection #2 solid triangle, 2021 = solid circle) and Somersby (open triangle) for all water types test. Dashed line indicates NEC value. For 2-d plot, data points are not shown for Somersby cyclopoids where 100% survival was observed for all concentrations tested up to 80 mg Ba/L (note that Somersby data points were incorporated into the NEC modelled curve fit).

**Table S12.** Dissolved (<0.45 µm filtered) barium toxicity values for Wellington (collected in 2021 and 2022) and Somersby cyclopoids (collected in 2020)

| Exposure (d) | Toxicity value, mg/L, dissolved barium* | | | | Curve-fit model^ | NEC^ |
| --- | --- | --- | --- | --- | --- | --- |
|  | EC50 | EC20 | EC10 | EC5 |  |  |
| 28 | 14 (7.9-20) | 6.1 (1.4-11) | 4.0 (0-8.2) | 2.8 (0-6.4) | Log-normal | 3.1 |
| 21 | 22 (17-28) | 9.5 (5.5-13) | 6.0 (2.7-9.4) | 4.2 (1.3-7.0) | Log-normal | 3.1 |
| 14 | 38 (32-45) | 17 (12-22) | 11 (6.3-16) | 7.8 (3.7-12) | Log-normal | 2.8 |
| 7 | 60 (48-72) | 35 (24-46) | 27 (15-38) | 21 (9.7-32) | Log-normal | 26 |
| 4 | 80 (62-98) | 48 (32-63) | 35 (19-51) | 27 (11-43) | Log-logistic | 32 |
| 2 | 127 (120-135) | 89 (80-97) | 73 (64-83) | 63 (53-73) | Log-normal | 179 |

* Dissolved barium was calculated as the 28-d average (i.e. dissolved barium concentrations remined stable throughout the toxicity test duration). Values in parentheses are 95% confidence limits.

^ Sigmoidal and no-effect curve (NEC) curve fits were applied to the combined data sets. NECs are preferred when concentration-response relationship show a threshold/tipping point.
